# The Psychology of COVID-19 Booster Hesitancy, Acceptance and Resistance in Australia

**DOI:** 10.3390/vaccines11050907

**Published:** 2023-04-27

**Authors:** Sabina Kleitman, Dayna J. Fullerton, Marvin K. H. Law, Matthew D. Blanchard, Rachel Campbell, Margaret-Ann Tait, Jennifer Schulz, Jihyun Lee, Lazar Stankov, Madeleine T. King

**Affiliations:** 1 School of Psychology, Faculty of Science, University of Sydney, Sydney, NSW 2006, Australia; dayna.fullerton@sydney.edu.au (D.J.F.); marvin.law@sydney.edu.au (M.K.H.L.); matthew.blanchard@sydney.edu.au (M.D.B.); r.campbell@sydney.edu.au (R.C.); margaret-ann.tait@sydney.edu.au (M.-A.T.); lazar.stankov@sydney.edu.au (L.S.); madeleine.king@sydney.edu.au (M.T.K.); 2 Sydney Nursing School, Faculty of Medicine and Health, University of Sydney, Sydney, NSW 2006, Australia; 3 Faculty of Law and Justice, University of New South Wales, Sydney, NSW 2052, Australia; jennifer.moore@unsw.edu.au; 4 Faculty of Medicine and Health, University of New South Wales, Sydney, NSW 2052, Australia; 5 School of Public Health, Faculty of Health and Environmental Sciences, Auckland University of Technology, Auckland 0627, New Zealand; 6 School of Education, Faculty of Arts, Design and Architecture, University of New South Wales, Sydney, NSW 2052, Australia; jihyun.lee@unsw.edu.au

**Keywords:** COVID-19, booster hesitancy, latent profile analysis, vaccine hesitancy, public health policy

## Abstract

COVID-19 booster vaccinations have been recommended as a primary line of defence against serious illness and hospitalisation. This study identifies and characterises distinct profiles of attitudes towards vaccination, particularly the willingness to get a booster dose. A sample of 582 adults from Australia completed an online survey capturing COVID-related behaviours, beliefs and attitudes and a range of sociodemographic, psychological, political, social and cultural variables. Latent Profile Analysis (LPA) identified three subgroups: Acceptant (61%), Hesitant (30%) and Resistant (9%). Compared to the Acceptant group, the Hesitant and Resistant groups were less worried about catching COVID-19, used fewer official COVID-19 information sources, checked the news less, were lower on the agreeableness personality dimension and reported more conservatism, persecutory thinking, amoral attitudes and need for chaos. The Hesitant group also reported checking the legitimacy of information sources less, scored lower on the openness to new experiences personality dimension and were more likely than the Resistant and Acceptant groups to report regaining freedoms (e.g., travel) and work requirements or external pressures as reasons to get a booster. The Resistant group were higher on reactance, held more conspiratorial beliefs and rated their culture as being less tolerant of deviance than the Hesitant and Acceptant groups. This research can inform tailored approaches to increasing booster uptake and optimal strategies for public health messaging.

## 1. Introduction

Vaccinations for coronavirus disease 2019 (COVID-19) have been an important strategy for developing widespread immunity to prevent severe illness and deaths, reducing the burden on healthcare systems and allowing societies and economies to reopen. The World Health Organization (WHO) reported that COVID-19 vaccinations prevented an estimated 19.8 million deaths globally in the first year of rollouts [1]. Globally, as of 10 April 2023, there had been 762,791,152 confirmed COVID-19 cases, 6,891,025 deaths and 13,340,275,492 vaccine doses administered [2]. Amid waning protection from primary doses and the rise of new variants, there is a need for widespread uptake of booster doses to maintain immunity and prevent serious illness and hospitalisation. This research provides valuable insights to inform public health policy and messaging to improve booster uptake for COVID-19 and potentially other diseases.

Previous research has conceptualised vaccination intentions and behaviours as ranging from complete acceptance to total refusal, and those holding varying degrees of uncertainty or hesitancy in between [3,4,5]. Understanding what drives people’s behaviours, intentions and attitudes towards the COVID-19 booster doses can help to inform effective, tailored approaches to promoting booster uptake by addressing underlying reasons or concerns and removing barriers, particularly in those who are undecided or ambivalent [6,7]. 

This research was conducted within the Australian context. Although Australia adopted an aggressive suppression policy, followed by an aggressive vaccination strategy (see below), the policies around booster vaccination (also described below) were similar to those of other countries around the world, including the United States, the United Kingdom, Canada and many European countries. This research, therefore, has global significance and can provide valuable lessons for managing COVID-19 and future pandemics. It may also help guide research and public health policies on other diseases that require ongoing vaccination, although implementation of such lessons may need to be tailored for specific diseases, countries and communities to achieve maximum efficacy.

The remainder of this introduction is structured as follows. In Section 1.1, Section 1.2 and Section 1.3, we review relevant literature to provide background, motivation and inspiration for the study we report in this paper. In Section 1.4 we state our objectives and hypotheses and highlight how our study adds to the evidence base about booster uptake.

### 1.1. Background and Context of COVID-19 Vaccinations in Australia

During the first 18 months of the pandemic, Australia took an ‘aggressive suppression’ approach, implementing border closures, travel bans, widespread social-distancing measures, contact tracing, stay-at-home orders and mandatory isolation and quarantine [8]. This approach succeeded in suppressing case numbers, allowing greater freedom outside of lockdowns and minimising COVID-19 deaths compared to other developed countries [9]. However, this success had a downside: complacency in the rollout of a vaccination strategy. When the Delta variant emerged in June 2021, only around 8% of the population aged 16+ were fully vaccinated [10]. This led to a surge in infection rates and the focus of the national COVID-19 strategy turned to increase vaccination rates.

By late 2021, Australia had administered more than 60 vaccines for every 100 people, catching up to Israel which had led an impressive vaccination campaign [11]. By mid-December 2021, more than 90% of the population aged 16+ were double-dose vaccinated, triggering the end of lockdowns and the easing of restrictions [12]. Around the same time, the highly transmissible Omicron variant reached Australia, leading to all-time high national case rates, with an average of 67,663 new cases reported each day, mid-January 2022 [13]. This figure is likely an underestimation, given the collapse of the testing system at the time [14]. At this point, the Australian Government decided it would no longer enforce public health protections; instead, Australia would go ‘forward to live with this virus with common sense and responsibility’ [15]. 

Australia began its COVID-19 booster vaccination rollout in November 2021. By mid-December, approximately 5% of the population aged 16+ had received a third (i.e., ‘booster’) dose [12]. Australian Medical Association President, Dr. Omar Khorshid, urged that amid the spread of Omicron, ‘three vaccine doses [are] essential for maximum protection in adults’ [16]. This comment was supported by a study of over two million adults in Sydney during the peak of the first Omicron wave, which reported that receiving a recent booster dose improved protection against serious illness by about 65% compared to a recent second dose [17]. Data based on self-reports from the United Kingdom also found that a booster dose reduced the likelihood of long COVID-19 symptoms [18]. 

### 1.2. Vaccine Intentions

Differences in the vaccine intentions of recipients before and during the emergency phases of the pandemic are well documented, ranging from complete acceptance to outright refusal and varying degrees of hesitancy [3,4,5,19]. In the present study, we focused on nonmandatory COVID-19 booster vaccination intentions, particularly hesitancy. Our focus on hesitancy was motivated by two main factors. First, public policies and interventions (through promoting booster uptake) may have had the greatest impact on those who were undecided or ambivalent compared to those who were adamant refusers [7,19,20]. Second, given the vaccination situation and public health policies in Australia at the time of data collection, only a small proportion of people remained vaccine resistant, whilst reluctance to receive a booster was increasing [21]. Additionally, Australia’s initial booster uptake was slow [22]. Thus, research clarifying the complex nature of booster hesitancy is likely to be of greater use to current and future public health policies in Australia and abroad. The broad objective of our study was to determine the psychological profiles of subgroups with differing attitudes towards COVID-19 booster doses. However, the existing literature on booster hesitancy is scarce. For this reason, we reviewed the broader literature on vaccination intentions, in addition to the literature on booster hesitancy to formulate our hypotheses. 

### 1.3. Factors Associated with COVID-19 Vaccine and Booster Hesitancy

#### 1.3.1. Sociodemographics

In a 2022 study of 3472 Australians, lower booster uptake was associated with younger age, being female, living in a disadvantaged area and not having completed Year 12 (the final year of high school in Australia), while higher booster uptake was associated with holding a postgraduate degree and being in the top-income quintile [21]. Studies from other countries produced similar results, with lower education [23,24,25,26,27], younger age [24,25], being female [24] and lower socioeconomic status [25] being associated with greater booster hesitancy. To further examine these findings, our study included age, sex, education level and financial situation. 

#### 1.3.2. Beliefs and Attitudes about COVID-19

Acceptance of a COVID-19 vaccine has been linked to perceived levels of effectiveness or benefits of a booster [23,24,26,28], while hesitancy is associated with low levels of concern about or perceived risk of being infected with COVID-19 [25,26], greater concern about adverse reactions or risks associated with the vaccine [23,26,28] and lower perceived need for a booster [26,28]. Lower support for nonpharmaceutical protective measures has also been linked to antivaccination beliefs and intentions [25,29]. This may be related to distrust in government authorities; for instance, one study of people from 19 countries found that higher levels of trust in government and institutions led to greater acceptance of the measures they endorsed [30].

In our study, we examined a range of beliefs surrounding COVID-19 protective measures and vaccinations, COVID-related concerns regarding infection, infringement of civil liberties and impact on the economy, personal finances and societal infrastructure (e.g., the healthcare system being overloaded, shortage of groceries and medical supplies) motivations towards complying with public health regulations during the emergency stage of the pandemic, trust in government bodies, and trust in science and health professionals.

Pandemic fatigue has also been considered a potential hurdle for vaccine uptake. The WHO describes pandemic fatigue as a response to the sustained pandemic situation involving distress, hopelessness and demotivation to continue following rules and recommendations [31]. This may manifest as reduced adherence to rules and less frequent information seeking and has been linked to the lower adoption of protective behaviours, including social distancing, hygiene behaviours and mask wearing [32]. Emerging research also indicates that increased pandemic fatigue may play a role in slowing booster uptake [33]. For instance, Bodas et al. [33] found that factors previously associated with greater COVID-19 vaccine uptake, including the perceived threat of COVID-19 and trust in authorities, were no longer predictive of booster vaccine uptake. They concluded that this could be explained by an increase in pandemic fatigue such that people were no longer as fearful of or concerned about COVID-19 and thus factors that previously motivated people to get a COVID-19 vaccine no longer did so. Consistent with this idea, another study from Malaysia found that those with lower pandemic fatigue exhibited greater willingness to receive a COVID-19 booster vaccine [34]. 

#### 1.3.3. Information Consumption, Perceived Health Literacy and Related Factors

Information consumption played a major role in people’s tendencies to comply with regulations early in the pandemic [35]. Misinformation can be a major hurdle for prompt COVID-19 vaccination; an experiment that exposed groups to either factual information or misinformation about both COVID-19 and vaccines found that misinformation lowered intentions to get a COVID-19 vaccine [36]. The use of different types of information sources and frequency of consumption have also been investigated in COVID-19 vaccine hesitancy research [5,21,37]. In British and Irish samples, Murphy et al. [5] found that the vaccine-resistant group consumed less information from newspapers, television, radio and government agencies than the acceptant group, and more from social media compared to the acceptant group, while the hesitant group did not differ from the acceptant group in its use of sources. By contrast, in Australia, Biddle and Sollis [21] found few associations between the use of different sources and booster uptake; only radio and television were associated with a slightly higher probability of booster uptake compared to other sources, which included professional advice, family or friends, newspapers or magazines and social media. They did, however, find a strong association between not getting COVID-19 information from any source and a lower probability of booster uptake. Another study, on Australian migrants, also found no differences in the sources of information used between those willing to get a COVID-19 booster and those who were uncertain [37]. To investigate these relationships further, our study examined people’s information-seeking behaviour. This included the frequency of using different COVID-19 information sources, checking news about COVID-19, sharing COVID-19 news with others and checking the legitimacy of information sources. The last was shown to be an important factor in compliance with government regulations during the first wave of the pandemic, whereby a noncompliant group was found to check the legitimacy of the information source less than a compliant group [35].

Another important factor was how people appraised and understood health information. This can be broadly captured by ‘health literacy’, defined as ‘the capacity to obtain, process and understand basic health information and services needed to make appropriate health decisions’ [38] (p. vi). Recent research found that better health literacy was associated with greater acceptance of a COVID-19 vaccine [39,40,41], perhaps because those with lower health literacy were more susceptible to believing misinformation. We also captured ‘bullshit receptivity’. That is, the tendency to perceive pseudo-profound statements as meaningful [42]. Pennycook et al. [42] proposed that this tendency may arise through two potential mechanisms: a cognitive bias towards believing things to be true, and an inability to detect false or meaningless information and consequently judging that which they cannot comprehend as being meaningful. People who score high on this tendency might thus be more susceptible to believing vaccine-related misinformation, impacting their vaccine beliefs and intentions.

#### 1.3.4. Psychological Characteristics

Although social and practical barriers played an important role in vaccine uptake [8], some personality characteristics have also been identified in the literature as important factors. Agreeableness and conscientiousness have been found to be associated with intentions to accept a COVID-19 vaccine [5,43,44], whereas higher neuroticism has been linked to an unwillingness to receive a COVID-19 vaccine [5,43] and lower openness and conscientiousness with hesitancy towards COVID-19 vaccines [43]. However, as these studies were conducted in 2020 based on intentions to receive primary COVID-19 vaccine doses, it is unknown whether the same traits are associated with a willingness to get a booster dose. 

To date, few studies have examined the relationship between cognitive ability and COVID-19 vaccine hesitancy. Murphy et al. [5] found that those who were hesitant or resistant to a COVID-19 vaccine scored lower on the Cognitive Reflection Test. This test is thought to be a measure of analytical thinking and the ability to override an initial intuitive response to arrive at the correct response [45]. However, more recent studies suggest this test may also capture numerical ability [46,47]. One study found that lower levels of generalised intelligence, derived from scores on a range of cognitive tests, were associated with greater COVID-19 vaccine hesitancy [48]. Again, this study was conducted at an earlier stage of the pandemic when people had not yet received even primary vaccinations. Thus, whether a relationship between COVID-19 vaccine hesitancy and cognitive ability and decision-making held during the later phases of the pandemic, when a large majority have received at least two doses, was yet to be investigated. Given these emerging findings, we included the Cognitive Reflection Test to capture analytical thinking and decision making, and the Esoteric Analogies Test as a short measure of verbal reasoning, which captures both fluid and crystallised intelligence [49]. 

Several other psychological characteristics have also been investigated in the COVID-19 literature. The disposition to experience psychological reactance, a motivational state that arises when an individual feels a threat or loss to their individual freedoms [50], was shown to have strong links to noncompliance with COVID-19 protective measures [35]. A pre-COVID study of 24 countries reported that higher dispositional reactance was associated with general antivaccination attitudes [51]. In the context of COVID-19 vaccine mandates, two studies concluded that mandates were detrimental to uptake as they elicited reactance and consequent vaccination resistance [52,53], while another concluded that mandates were not associated with greater reactance [54]. The relationship between reactance and non-mandated COVID-19 booster vaccines had not yet been investigated, warranting examination in our study.

Vaccine hesitancy is also associated with concerns about adverse side effects and risks associated with the vaccine [23,26,28], previous negative reactions to vaccines [55] and uncertainty about the long-term consequences and benefits of the new COVID-19 vaccines [28]. Although these factors are known to be important in vaccine hesitancy [23,26,28,55], our research focused on psychological characteristics related to perceptions of uncertainty associated with a novel vaccine. Intolerance of uncertainty is a disposition associated with excessive worry about the possibility of negative outcomes, irrespective of the probability of such outcomes [56]. Individuals high in this trait may have greater anxiety and worry about the effects of vaccines and thus greater hesitancy towards receiving vaccinations. Supporting this premise, McNeil and Purdon [57] found that intolerance of uncertainty was related to greater hesitancy towards vaccination in those without an anxiety disorder, but the inverse was the case for those with an anxiety disorder. Factors related to vaccine hesitancy, including mistrust in vaccine efficacy and experiencing vaccination side effects, have been shown to affect psychological stress related to getting a vaccination [58]. Moreover, COVID-related burnout has been found to be connected to a lower intention to receive a COVID-19 booster vaccination [59]. Psychological resilience, or the capacity to withstand stress, has been associated with less COVID-related burnout and a direct, positive relationship with the intention to get a booster vaccination [59]. Accordingly, in our study, we included measures of psychological resilience and intolerance of uncertainty to further examine these relationships.

#### 1.3.5. Political, Cultural and Social Attitudes

In addition to individual traits and behaviours, people’s attitudes and views of their political, cultural and social context are important in the context of the pandemic. In research from the United States, more conservative social and political views were associated with hesitant and antivaccination attitudes [23,29,60], though not with intentions to get a booster [23]. To our knowledge, the relationship between conservatism and booster intentions in Australia was yet to be investigated and thus was examined in this study. 

As previously mentioned, trust in the government plays a role in how regulations and recommendations from public health authorities are received by the public. There is evidence this may play an important role in vaccine uptake. Previous studies reported that lower confidence and trust in the government and information given by public health/government agencies were related to vaccine and booster hesitancy [27,61]. Freeman et al. proposed a set of beliefs and perceptions that lead to mistrust in government and institutions and influence COVID-19 vaccine uptake [62]. They included conspiracy and paranoid beliefs, which have been shown to be associated with vaccine hesitancy and resistance [5,44,62,63]. Freeman et al. also identified expressing a ‘need for chaos’, which reflects discontent with current political and social structures [62]. They found that these beliefs, along with other perceptions of healthcare authorities, formed a higher-order mistrust factor that predicted vaccine hesitancy [62]. The role of trust in booster hesitancy in Australia had yet to be examined; our study fills this gap and investigates whether the need for chaos, conspiracy beliefs and paranoia relate to mistrust and booster uptake. 

Perceptions of cultural tightness have been investigated in relation to behavioural decisions about COVID-19. In ‘loose’ cultures, freedom is highly valued, whereas ‘tight’ cultures have strict norms and a low tolerance for deviance [64]. In a study of participants from Australia, Canada, the United States and the United Kingdom, Kleitman et al. found that people who rated their culture as looser reported lower compliance with COVID-19 protective measures in the early stages of the pandemic compared to those who rated their culture as tighter [35]. Ng and Tan [65] examined the relationships between willingness to receive a COVID-19 vaccination and the level of cultural tightness in 15 countries. Contrary to their expectation, they found that greater perceptions of looseness were associated with a greater willingness to vaccinate. They surmised that this may have been because the virus was better controlled in tighter cultures and thus posed less risk and hence less urgency to receive a vaccine. Additionally, their data were collected between late 2020 and early 2021 when vaccination programs had not yet been rolled out, and there were greater concerns about risks associated with the vaccine, which in some countries may have been perceived to outweigh the risk of COVID-19 [65]. 

Pandemics present choices about morality; individuals are called to act, not only to protect themselves but for the collective good, eliciting a moral obligation to follow rules and recommendations from authorities. Altruism is characterised by showing care for and a desire to help others, whereas amorality is associated with self-interested attitudes i.e., a lack of altruism. Previous research found that higher amorality scores were associated with lower compliance with COVID-protective measures [35], using a measure that captured disregard for others, for rules and for social values. We included this measure of amorality in the current study to examine whether this relationship holds for vaccination behaviours, as found by Murphy et al. [5], whereby altruism was lower in vaccine-hesitant and vaccine-resistant groups compared to acceptant groups. 

### 1.4. The Present Study

The research reviewed above provides insight into some of the characteristics and attitudes that may differ among those who are acceptant, hesitant and resistant to COVID-19 vaccines, though to a lesser extent, of nonmandatory booster doses. This study builds on previous research that investigated vaccine acceptance and hesitancy, examined a range of factors during the phase of the pandemic in Australia when many regulations had been lifted, a large majority of the population had received their primary doses, and booster doses were recommended as an important line of defence. 

Given that research into factors associated with attitudes towards booster vaccines is needed to inform evidence-based public health policies relevant to various stages of a pandemic, we adopted a person-centred approach that enabled us to identify groups who shared similar vaccination attitudes. Our study was designed to extend the existing literature by integrating this person-centred approach with variable-centred methods to examine group differences in a broad range of factors expected to be associated with booster uptake. This novel dual approach was adopted to provide a comprehensive picture of the multifaceted and complex factors underlying COVID-19 booster uptake. 

#### Aims and Hypotheses

The dual aims of the study were: (1) to identify subgroups classified by focal variables, including willingness to receive a booster, COVID-19 vaccination beliefs, beliefs about COVID-19 protective measures, pandemic fatigue, trust in authorities and information sources, concerns about COVID-19 and the pandemic in general, financial comfort, education level and health literacy; (2) to examine differences between identified subgroups on a comprehensive suite of variables, including vaccination status at the time of our survey and a range of personal characteristics (specifically demographic, psychological, cognitive ability and decision-making, political and cultural characteristics). 

In line with previous research, we hypothesised that there would be groups who systematically differed in their willingness to receive a COVID-19 booster. Specifically, we expected a group who were acceptant of vaccinations, had the highest rates of COVID-19 vaccinations, had a high degree of trust in government, science and health practitioners, greater compliance with and support for protective measures, belief in the benefits of vaccines and higher concerns about being infected with COVID-19. We also expected to find a group who showed hesitancy and may have delayed receiving a booster or were still reluctant, and another group who refused to get a booster had the lowest rates of vaccination and held negative views towards COVID-19 vaccinations, protective measures, government, science, and health professionals. In line with previous research, we used the labels Acceptant, Hesitant and Resistant for these hypothesised groups. Further, we expected that information consumption behaviours, psychological characteristics and political and cultural attitudes would differ between the identified profiles. We expected that the Hesitant and Resistant groups would be younger, consist of more females and have lower education levels. 

In relation to COVID-19 beliefs, we hypothesised that the Hesitant and Resistant groups would also report less support for other protective measures, hold more antivaccination beliefs and exhibit greater pandemic fatigue. We also hypothesised that the Hesitant and Resistant groups would consume less information about COVID-19 in general, less information from official government sources and more from casual, unofficial sources, and have a lower tendency to check the legitimacy of the source, while the Acceptant group would use more official sources, would tend to check the legitimacy of the information sources more and would have greater health literacy. 

In relation to the psychological factors, we hypothesised that greater booster uptake (Acceptant) would be associated with higher agreeableness, conscientiousness and openness/intellect personality dimensions and resilience, while lower uptake (Hesitant and Resistant) would be associated with lower scores on the Esoteric Analogies and Cognitive Reflection tests, greater psychological reactance and greater intolerance of uncertainty. 

In relation to the political, social and cultural variables, we expected that hesitancy and resistance would be associated with higher distrust towards government, science and health professionals, with more conspiratorial and paranoid beliefs, a higher need for chaos, perceptions of culture as looser and higher amorality. 

## 2. Materials and Methods

### 2.1. Participants and Recruitment

Adults (N = 598) living in Australia were recruited to participate in this study via an online research platform, Prolific (https://www.prolific.co/, accessed on 11 January 2022). An advertisement was published on Prolific stating that we were interested in people’s behaviours and attitudes as we move towards ‘living with COVID-19’ and that participants would be required to complete a survey (approximately one-hour long) consisting of biographical and health-related questions, cognitive ability and decision-making tasks, questions about perceptions and behaviours related to the COVID-19 pandemic, and about personality characteristics. Eligible participants were those over 18 years old, residing in Australia, and with sufficient English language proficiency. Eligible Prolific users could view the advertisement on their dashboard and, if interested, opt in. Prolific also notified a random subset of eligible individuals via email of the study; if interested, those individuals could opt in. Participants were compensated £7 (approximately $13AUD) for their time. This survey was part of a larger research project; only aspects relevant to the stated aims of this paper are reported in this paper. Ethics approval was granted by the University of Sydney Human Research Ethics Committee (protocol number 2021/971).

Data were collected from 27 January to 10 March 2022. In the lead up to this period, Australia experienced an unprecedented spike in COVID-19 cases as the highly transmissible Omicron variant spread, ending two years of successful suppression. The 7-day average of new cases went from approximately 1300 to 2100 in early to mid-December 2021 to a peak 7-day average of 109,215 in mid-January 2022 [66]. See Supplementary Materials (Table S1) for a list of public health orders that were in place, lifted or implemented during the data collection period. 

### 2.2. Measures

Table 1 summarises all the measures employed in this study, including the number of items, the response scale, the score range and the direction. Validated measures were used to capture pandemic fatigue, COVID-19 conspiracy beliefs, health literacy, personality, cognitive ability, decision making, resilience, bullshit receptivity, reactance, conservatism, conspiracy mentality, persecution, need for chaos, cultural tightness and amorality. 

In addition to using validated measures and where existing measures were not available, new study-specific measures were developed to capture COVID-19 behaviours and attitudes. Most of these measures were originally developed by the authors for studies conducted earlier in the pandemic [35,67]; however, given the rapidly changing nature of the pandemic, we modified and adapted these to maintain their relevance in the context of the pandemic at the time of data collection. Items and scoring for measures developed by the authors (study-specific measures) are provided in Supplementary Materials. Exploratory factor analyses (EFA) were performed to identify the underlying dimensions of these measures; the results are provided in Supplementary Materials (Tables S2–S9). 

**Table 1:** Measures employed in this study.

Measure (Authors)	Number of Items and Response Scale	Scoring	Dimensions and Example Items	Internal Consistency (Previous Studies)
**Demographics**				
Financial comfort	1 item Sliding scale from 0 *(not at all comfortable)* to 100 *(very comfortable)*	0–100	‘How would you rate your level of financial comfort currently?’	-
Education level	1 item 1 = year 11 or below 2 = year 12 3 = trade certificate/apprenticeship 4 = diploma 5 = bachelor’s degree 6 = higher degree	1–6	“What is the highest level of education you have completed?’	-
**COVID-related variables**				
Vaccination status	1 item 0 = no doses 1 = one dose only 2 = two doses only [or three doses as primary course if immunocompromised] 3 = primary course and booster	0–3	‘Have you received a COVID-19 vaccination?’	-
Booster willingness	1 item 1 = definitely not 2 = probably not 3 = unsure 4 = probably 5 = definitely 6 = have already had booster	1–6 Higher = more willing	‘When a COVID-19 vaccine booster is available to you to boost your protection against COVID-19, will you get it?’	-
COVID-19 Beliefs (study specific, see Supplementary Materials)	24 items (1) *strongly disagree* to (5) *strongly agree*	Factor scores (Bartlett Method) Higher = greater endorsement	Efficacy of protective measures e.g., ‘Social distancing is effective in slowing the spread of COVID-19’ Anti-regulations e.g., ‘Mandatory COVID-19 vaccination requirements violate my civil rights’ Support for alternative measures e.g., ‘alternative medicine like consuming certain teas, herbs, minerals, or supplements are good preventative measures for COVID-19’	-
Vaccination Attitudes Examination Scale (modified) [68]	13 items (1) *strongly disagree* to (6) *strongly agree*	Mean scores Higher = less support for vaccinations	Mistrust of vaccine benefit e.g., ‘I feel there are safe COVID-19 vaccines available’ (R) Worries about unforeseen future effects e.g., ‘There may be problems with the COVID-19 vaccines that we have not yet discovered’ Concerns about commercial profiteering e.g., ‘COVID-19 vaccines make a lot of money for pharmaceutical companies, but do not do much for regular people’ Preference for natural immunity e.g., ‘Natural immunity lasts longer than a COVID-19 vaccination’	0.80 to 0.93 [68]
Multidimensional COVID-19 Worry Index (study specific, see Supplementary Materials)	19 items (1) *never* to (4) *always*	Mean scores Higher = more worried	Concerns about catching COVID-19 and infrastructure e.g., ‘I am concerned about the health of my family members due to COVID-19’ Concerns about political systems, social liberties and economy e.g., ‘I am worried about political unrest in Australia’ Financial concerns e.g., ‘I am anxious about losing money due to COVID-19’	-
COVID-19 Readiness for Lockdown (study-specific, see Supplementary Materials)	7 items (1) *strongly disagree* to (5) *strongly agree*	Mean score Higher = more ready	‘Should another lockdown be needed, I will follow the rules’	-
Compliance Attitudes (study specific, see Supplementary Materials)	5 items, repeated for two time periods: currently and before December 2021 Slider scale from (0) *does not apply at all* to (100) *applies very much*	Mean score Higher = more compliant with and understanding of rules/recommendations	‘I adhere to the current COVID-19 rules or recommendations’	-
Reasons for getting vaccinations (study specific, see Supplementary Materials)	10 items, repeated for primary vaccination (for those who had received at least two doses), and booster (either relating to reasons one got a booster for those who had received a booster dose, or why one would receive a booster if not yet done so) (1) *strongly disagree* to (6) *strongly agree*	Mean scores Higher = greater endorsement of reason	To protect self and others e.g., ‘I got vaccinated to protect the greater community’ Due to pressure from others e.g., ‘I got vaccinated due to pressure from my family/friends’ To regain freedoms, travel and required for work e.g., ‘I got vaccinated to regain freedoms (e.g., socialising, hospitality, events)’	-
Pandemic Fatigue Scale [32] and Neglect from the Brief Pandemic Fatigue Scale [69] (The Boredom subscale from the Brief Pandemic Fatigue Scale was not included due to its substantial overlap with the Informational Fatigue subscale.)	9 items (1) *strongly disagree* to (7) *strongly agree*	Overall mean score (an exploratory factor analysis was run on the items. All items converged and loaded on a single factor; thus, an overall mean score was calculated based on these results.) Higher = more fatigued	Pandemic Fatigue Scale: Informational fatigue: ‘I am sick of hearing about COVID-19’ Behavioural fatigue: ‘I am losing my spirit to fight against COVID-19’ Brief Pandemic Fatigue Scale: 3. Neglect: ‘I am already so tired of the COVID issue that I am not as careful as I was at the beginning’	0.83 to 0.86 [32] 0.85 [69]
Trust (study specific see Supplementary Materials)	15 items (1) *strongly distrust* to (5) *strongly trust*	Mean scores Higher = greater trust	Trust in Science and Health Professionals e.g., doctor, scientific articles Trust in Government e.g., official Government/health organisation websites, Trust in Unofficial Sources e.g., family or friends, podcasts, social media Trust in Media e.g., mainstream news (TV, radio, internet), mainstream media	-
OCEANS Coronavirus Conspiracy Scale [62]	7 items (1) *do not agree* to (5) *agree completely*	Mean score Higher = greater belief in conspiracies	‘The virus is a hoax’	0.94 [62]
Information Sources (study-specific, see Supplementary Materials)	10 items (1) *never* to (5) *all of the time*	Mean scores Higher = more frequent use of sources	Official sources e.g., ‘Official Government/health organisation websites’ Unofficial sources e.g., ‘social media’ ‘family or friends’	-
Check News	1 item (1) *never* to (5) *all of the time*	Higher = more frequent	‘How often do you check the news regarding COVID-19?’	-
Source Check	1 item (1) *never* to (5) *all of the time*	Higher = more frequent	‘How often do you check the legitimacy of the source of information about COVID-19?’	-
Share News	1 item (1) *never* to (5) *all of the time*	Higher = more frequent	‘How often do you share news about COVID-19 with family/friends?’	-
**Psychological Variables**				
All Aspects of Health Literacy Scale [70]	13 items	Overall summed score Higher = better health literacy	Functional: ‘How often do you need someone to help you when you are given information by your doctor, nurse, or pharmacist?’ Communicative: ‘When you talk to a doctor or nurse, do you ask the questions you need to ask?’ Critical: ‘How often do you try to work out whether information about your health can be trusted?’	0.75 [70]
Mini International Personality Item Pool [71]	20 items *(1) very inaccurate* to (5) *very accurate*	Summed scores Higher = higher on trait	Extraversion: ‘I am the life of the party’ Agreeableness: ‘I sympathize with others’ feelings’ Conscientiousness: ‘I get chores done right away’ Neuroticism: ‘I have frequent mood swings’ Intellect/Openness: ‘I have a vivid imagination’	0.65 to 0.82 [71]
Esoteric Analogies Test [72]	14 items	0–100%	‘FLAME is to HEAT as ROSE is to: (a) LEAVES; (b) SCENT; (c) THORN or (d) PETAL’	0.64 and 0.76 [73,74]
Cognitive Reflection Test [75]	4 items	0–100%	‘Jerry received both the 15th highest and the 15th lowest mark in the class. How many students are in the class?’	0.72 [75]
Connor-Davidson Resilience Scale Short [76]	10 items (0) *not true at all* to (4) *nearly always true*	Summed score Higher = more resilient	‘I can deal with whatever comes’	0.85 [76]
Intolerance of Uncertainty Scal [56]	12 items (1) *not at all characteristic of me* to (4) *entirely characteristic of me*	Summed score Higher = greater intolerance	‘Unforeseen events upset me greatly’	0.91 [56]
Bullshit Receptivity Scale [42]	10 items (1) *not at all profound* to (5) *very profound*	Mean score Higher = more receptive to pseudo-profound statements	‘Hidden meaning transforms unparalleled abstract beauty’	0.82 to 0.96 [42]
Hong Psychological Reactance Scale [77]	14 items (1) *strongly disagree* to (6) *strongly agree*	Mean score Higher = more reactance	‘Regulations trigger a sense of resistance in me’	0.75 to 0.80 [78]
**Political, Cultural, Social Attitudes**				
Social Conservatism Scale [79]	3 items were selected from the 12-item scale. (1) *fully disagree* to (5) *fully agree*	Mean score Higher = more conservative	‘We have to respect our history and tradition’	0.55 [37]
Conspiracy Mentality Questionnaire [80]	13 items (1) *strongly disagree* to (7) *strongly agree*	Mean score Higher = greater conspiracy mindset	‘There are many very important things happening in the world about which the public are not informed’	0.84 [81]
Persecution from the Persecution and Deservedness Scale [81]	10 items (10) *certainly false* to (5) *certainly true*	Mean score Higher = greater persecutory thinking	‘There are times when I worry that others may be plotting against me’	0.84 [81]
Need for Chaos [82]	7 items (1) *strongly disagree* to (7) *strongly agree*	Mean score Higher = greater need for chaos	‘I get a kick when natural disasters strike in foreign countries’	0.90 [82]
Cultural Tightness–Looseness [64]	6 items (1) *strongly disagree* to (6) *strongly agree*	Mean score Higher = perceive culture as tighter	‘There are many social norms that people are supposed to abide by in this country’	0.85 [64]
Amoral Social Attitudes [83]	6 items (1) *fully disagree* to (5) *fully agree*.	Mean score Higher = greater amorality	‘I hate obligations and responsibilities of any kind’	0.64 [35]

Participants were also asked to indicate their current COVID-19 vaccination status and completed a demographics questionnaire consisting of age, sex, financial comfort and the highest level of education completed. We also had a comprehensive section on the side-effects people experienced after receiving each dose of COVID-19 vaccination and the brand of vaccination received. A majority of the sample reported no or mild reaction, thus we were unable to capture severe post-vaccine side effects in sufficient numbers (for dose 1: severe = 1.5%. Dose 2: severe = 1.9%. Booster dose 1: severe = 1.1%. The number of participants who reported being diagnosed with the following serious side effects were as follows: thrombosis with thrombocytopenia syndrome = 2; blood clotting in areas other than heart or brain = 0; heart problems (e.g., myocarditis, pericarditis) = 3; neurological problems = 0; other = 0.). Thus, despite our intention to examine and control for severe side effects, these variables did not contribute meaningfully to our results. Similarly, we collected data on existing comorbidities (see Table S13 in the Supplementary Materials). A large majority of samples (78.5%) did not have any, and those who reported having them varied in different conditions, preventing us from investigating these matters meaningfully. These critical matters must be investigated in large-scale medical research.

### 2.3. Statistical Analyses

Latent profile analysis (LPA) is an increasingly popular mixture modelling method that identifies categorical subgroups based on patterns of values of predictor variables [84]. We selected LPA over other clustering methods due to its vast array of model specifications to determine the best fit and its probabilistic assignment to subgroups for each participant [85,86]. 

To identify subgroups within the current sample, LPA was conducted on 19 variables, including beliefs and attitudes surrounding COVID-19, trust towards existing entities, and health, financial and education characteristics. Following established methods, all variables were standardised to allow for comparison [86]. LPA was conducted for 1 to 6 class solutions and several fit indices were compared to determine the best-fitting profile solution. The terms ‘subgroup’, ‘class’ and ‘profile’ are used interchangeably hereafter. For AIC, BIC and SABIC, the best-fitting model would have the lowest value, while for BLRT and log likelihood, nonsignificant comparisons between the k and k − 1 profile solutions would indicate no statistically significant improvement in the k solution. In such a case, the prior (k − 1) solution was often chosen for the parsimoniousness of the optimal model. Finally, entropy indicates the quality of class separation, with higher values indicating better fit (for further descriptions of fit indices, see Spurk et al. [86]). 

Due to a high correlation between the COVID-19 beliefs measure and the vaccination attitudes examinations scale (r = 0.79), we ran an EFA on items from both measures to examine their convergence. The items converged into three factors, which were used in the subsequent analyses. These were labelled antivaccination beliefs, beliefs in protective measures and regulations, and beliefs in alternative measures. The full results of the EFA are provided in the Supplementary Materials (Table S2). 

**Figure 1:**
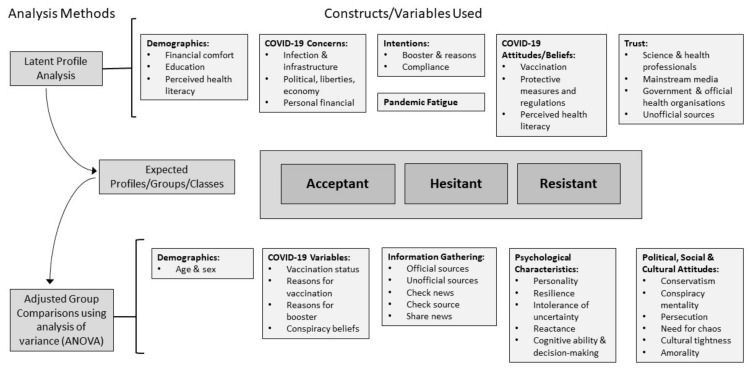
Summary of the variables and analysis methods used.

All resultant profiles were compared using ANOVAs and pairwise contrasts to determine differences in vaccination-related variables (e.g., vaccination status, reasons for getting vaccinated), in addition to psychological, political and cultural characteristics. Figure 1 presents a summary of the variables included and the analysis methods used. To account for unequal sizes of group membership and heterogeneity of variance across the three groups, comparisons between the three profiles for continuous variables were examined using Welch’s ANOVA and Games–Howell post hoc pairwise comparisons [87]. For categorical variables, chi-squared tests were conducted. Results were interpreted based on eta-squared (η^2^) following established guidelines with cut-off points of 0.01 as small, 0.09 as medium and 0.25 as large [88,89]. All analyses were conducted in R [90].

## 3. Results

### 3.1. Sample Characteristics

**Figure 2:**
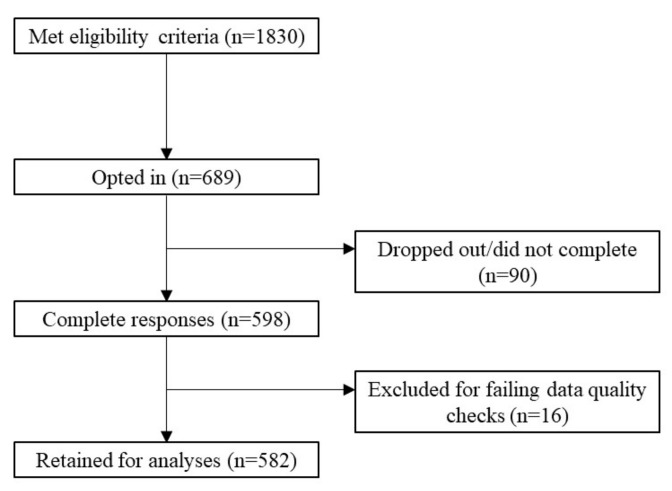
Respondent flow diagram.

Figure 2 shows the recruitment flow, response rates and exclusions (see Supplementary Materials for details of data cleaning protocol and exclusion criteria). The final sample of 582 Australian residents had a mean age of 34.68 (SD = 12.79); 58.2% were female and 41.8% male. Additionally, 30.1% of the participants lived in NSW, 28.4% in Victoria and 41.5% in other states/territories. About 2% of the sample reported they had completed Year 11 or below, 36% had completed Year 12, a trade certificate, or diploma, 39% had completed an undergraduate degree and 23% had completed a postgraduate degree. Table S13 in the Supplementary Materials presents more detailed sample characteristics alongside Australian general-population statistics. 

### 3.2. Profile Selection

**Figure 3:**
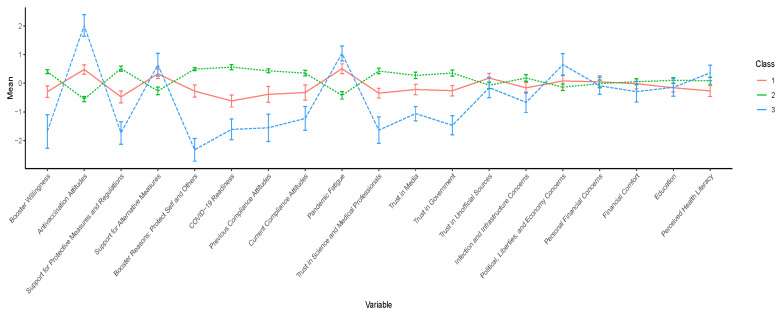
Variable standardized mean (z-scores) comparisons between the three profiles found within the 3-class solution. Higher = more on each variable. Class 1 Hesitant (n = 176), Class 2 Acceptant (n = 353), Class 3 Resistant (n = 53).

Table 2 summarises the fit indices for the six profile solutions. Most fit indices i.e., AIC, BIC, SABIC, BLRT and log likelihood, indicated that model fit was better for profiles with more classes, as was expected (i.e., improved data fit with a higher number of parameters). However, entropy was highest for two- and three-class solutions, being 0.94 and 0.91 respectively (entropy value for the one-class solution is one due to no required class classification and, thus, should not be compared with other class solutions). To determine the best fit between the solutions, we examined class classification accuracy, proportions and interpretability. Both solutions demonstrated good classification accuracy for all classes (above 90%), as well as good class proportions. Both solutions highlight two distinct profiles: a minority noncompliant, distrusting subgroup and a majority compliant and trusting subgroup. The three-class solution further highlighted a third profile with the most predictor variable values in between the two other profiles. This class reflected a ‘moderate’ subgroup, in between those with strong compliant and noncompliant beliefs and behaviours. The interpretation of this moderate subgroup was distinctive and consistent with the acceptant, hesitant and resistant subgroups found in previous vaccination research [4]. It also provided a greater opportunity for subsequent analyses to understand policy-relevant group differences between those who were acceptant, hesitant and resistant to COVID-19 vaccines/boosters. Therefore, the three-class solution was selected for further investigation. Standardised mean scores (z-scores) of the predictor variables for the three profiles found in the three-class solution are shown in Figure 3 below.

### 3.3. Interpretation of the Three-Class Solution

Percentages of participants in each profile were 30% for class 1 (n = 176) (represented by the red line), 61% for class 2 (n = 353) (represented by the green line) and 9% for class 3 (n = 53) (represented by the blue line) in Figure 3. 

To interpret the classes, we started with Class 3, which scored high on a range of vaccination-resistant tendencies, and hence was labelled the ‘Resistant’ group. This class exhibited noncompliant beliefs and behaviours towards COVID-related health measures, with the lowest booster willingness and regulation compliance and the highest antivaccination attitudes, pandemic fatigue and endorsement of unofficial alternative preventive measures (e.g., inhaling steam, taking supplements). As predicted, this class also exhibited low trust in science, media and government, low concerns about catching COVID-19 and high concerns about the effect of COVID-19 measures on political unrest, liberties and the economy. Finally, this class had the highest perceived health literacy. Post hoc analyses examining the three dimensions of the health literacy scale (functional, communicative and critical) revealed these differences were mostly due to the Resistant group scoring higher on the ‘critical literacy’ subscale (see Supplementary Materials, Table S13 for full results).

Class 2 captured the majority of participants, and this group reported the highest booster willingness, COVID-19 preparedness and compliance and the lowest antivaccination attitudes, hence was labelled the ‘Acceptant’ group. Class 2 also showed the lowest pandemic fatigue, lowest endorsement of alternative preventive measures, highest trust in science, media and the government, the highest concern about catching COVID-19 and the lowest concern about political liberties and the economy. This class scored moderately on perceptions of health literacy.

Finally, as predicted, a profile constituting the ‘moderates’ regarding COVID-related compliance beliefs and behaviours emerged (Class 1), exhibiting scores between the other two classes for almost all COVID-19 and trust-related factors. This profile was labelled the ‘Hesitant’ group. People comprising this group had moderate levels of booster willingness, regulation compliance, and trust towards science, media and the government. They also had moderate levels of antivaccination attitudes, pandemic fatigue, endorsements towards alternative preventive measures and concerns regarding catching COVID-19, liberties and the economy. Finally, this class exhibited the lowest perceived health literacy. 

We note that all three classes had similar levels of financial concerns/comfort and education levels, and these variables did not contribute to the separation of the three classes.

### 3.4. Profile Differences in Demographics and COVID-19 Variables

Table 3 summarises comparisons between the three profiles (classes) on demographics and COVID-19-related variables.

#### 3.4.1. Demographics

Significant sex differences were found between the three classes, *χ*^2^_2,N=572_ = 20.40, *p* < 0.001 (ten participants did not report their sex). The Resistant group (Class 3) were mostly male (60.4%). The Acceptant group (Class 2) were mostly female (65.4%), and the Hesitant group (Class 1) had a similar number of males and females (49.4% females). There was a small difference in age between classes, with the Hesitant group being somewhat younger than the Acceptant group (see Table 3). 

#### 3.4.2. COVID-19 Variables: Vaccination and COVID-19 Conspiracy Beliefs

The three classes differed strongly on vaccination status (number of doses received) at the time of the survey and on COVID-19 conspiracy beliefs, with all pairwise comparisons being significant. Consistent with expectations, the Resistant group had the lowest number of vaccination doses on average and the highest levels of conspiracy beliefs, while the Acceptant group had the highest number of vaccination doses and the lowest levels of conspiracy beliefs. For both variables, the Hesitant group scored in between the Acceptant and Resistant groups. 

**Figure 4:**
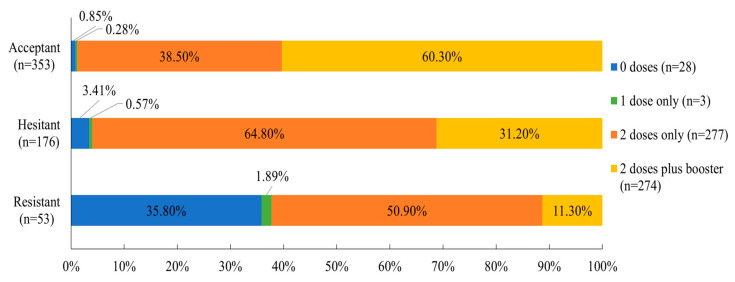
Proportions of COVID-19 vaccination doses received within each profile.

To further clarify differences in vaccination status, we examined the specific proportions of the number of doses received within each class (see Figure 4). Most members of the Acceptant and Hesitant groups (i.e., more than 96%) had received at least two doses of primary vaccination, with over 60% of the Acceptant group and 31% of the Hesitant group having received a booster. Comparatively, only 62% of the Resistant group had received two or more doses and more than a third (35.80%) had received no vaccinations at all. 

#### 3.4.3. Reasons for Getting Primary and Booster Vaccinations

**Figure 5:**
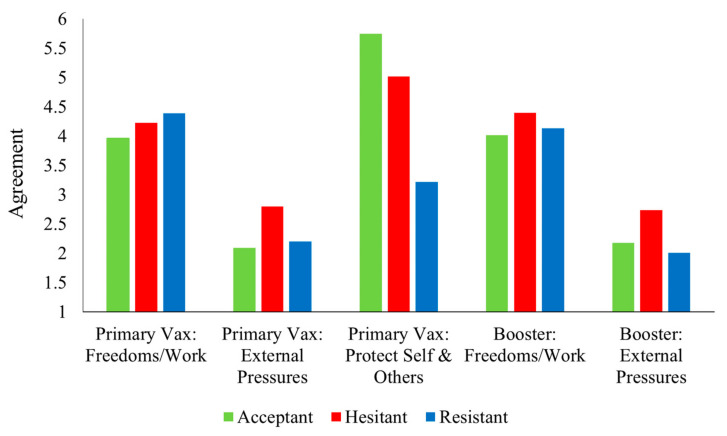
Mean scores of rated agreement (1 = strongly disagree; 6 = strongly agree) with reasons for getting primary and booster vaccinations for the three profiles (see Table 3 for statistical significance).

Table 3 and Figure 5 present differences in the reported reasons for getting primary and booster vaccinations. Figure 5 presents patterns for the reported reasons for vaccination for primary vaccination doses (554 respondents who had received primary vaccination doses) and boosters (274 who had already received a booster dose responded, based on why they got a booster and 277 primary-dose vaccinated respondents who had not yet received a booster responded based on why they would get a booster. For primary dose vaccinations, all three profiles differed significantly from each other in the degree to which they reported getting vaccinated to protect themselves and others, with the Acceptant group scoring highest and the Resistant group scoring lowest. The Hesitant group reported getting vaccinated due to ‘external pressures’ significantly more than both the Acceptant and Resistant groups for both primary dose vaccinations and boosters. The patterns differed for the ‘regaining freedoms and work requirements’ reason. There were no differences between groups for primary-dose vaccinations, though, for booster doses, the Hesitant group scored significantly higher than the Acceptant group. The Resistant group did not differ significantly from either of the other two groups for this booster reason. 

### 3.5. Profile Differences in Information Gathering

Table 4 presents the results of comparing the three profiles in terms of the information-gathering variables. There were large differences between the groups in the use of official sources for COVID-19 information. The Acceptant group reported using official sources significantly more than both the Hesitant and Resistant groups; the Hesitant and Resistant groups did not differ significantly from each other. For unofficial sources, there were no significant differences between groups. However, the Acceptant group reported checking the legitimacy of sources and sharing COVID-19 news with others significantly more than the Hesitant group but did not differ significantly from the Resistant group. The Acceptant group also reported checking the news about COVID-19 more frequently than the Hesitant and Resistant groups. 

### 3.6. Profile Differences in Psychological Measures

Comparisons of the three profiles on psychological measures, including cognitive ability and decision making, personality, as well as political, social and cultural variables are shown in Table 5.

#### 3.6.1. Psychological Characteristics and Cognitive Ability

Moderate differences were found for the Esoteric Analogies Test (EAT) accuracy and agreeableness personality score, wherein the Acceptant group scored higher than the Hesitant and Resistant groups. There was also a small but significant difference between the Acceptant and Hesitant groups on the openness personality dimension, with the Acceptant group scoring higher. The three profiles differed strongly and significantly on psychological reactance, with the Resistant group scoring the highest and the Acceptant group scoring the lowest. No other differences were found. 

#### 3.6.2. Political, Cultural and Social Attitudes

There were large differences between the profiles on amorality, need for chaos and conspiracy mentality. The Resistant group scored higher on amorality and conspiracy mentality than both the Acceptant and Hesitant groups and had a greater need for chaos compared with the Acceptant group. The Hesitant group scored higher than the Acceptant group on amorality, need for chaos and conspiracy mentality. 

There were moderate differences between the groups on conservatism, cultural tightness and persecution. The Hesitant and Resistant groups scored significantly higher on conservatism and persecution than the Acceptant group. Finally, the Resistant group perceived their culture to be tighter than both the Hesitant and Acceptant groups.

**Figure 6:**
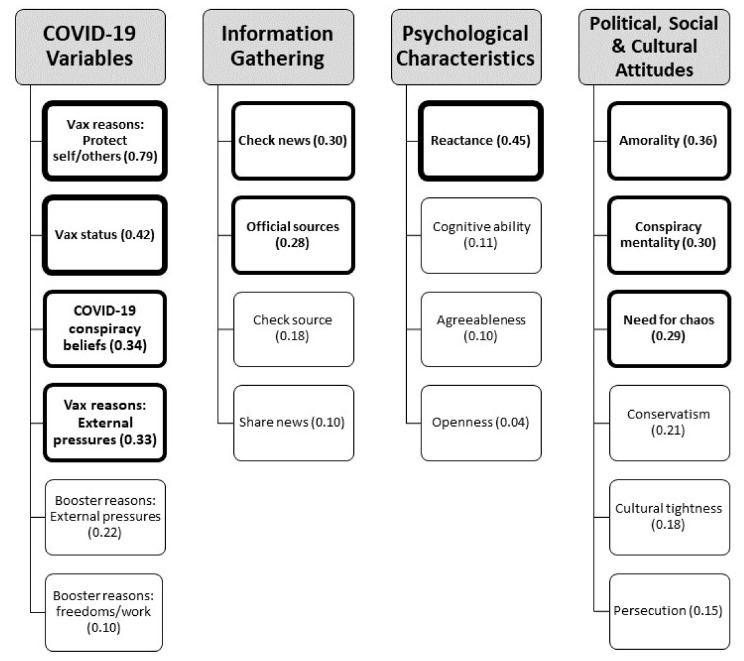
Summary of variables that yielded statistically-significant differences between profiles (Acceptant, Hesitant, Resistant). Effect size (η^2^) presented in parentheses. Border thickness reflects effect size: thin border = small η^2^ > 0.01; medium border = medium η^2^ > 0.09; thick border = large η^2^ > 0.25. Abbreviations: Coronavirus disease 2019 (COVID-19), vaccination (vax).

Figure 6 presents a summary of ANOVA results, illustrating variables with statistically significant differences between profiles. 

## 4. Discussion

This study synthesised a comprehensive selection of transdisciplinary theory-driven variables to better understand the complex nature of behaviours, attitudes and beliefs associated with COVID-19 booster behaviours and intentions. We identified three subgroups in our sample: Acceptant, Hesitant and Resistant. Individuals within each subgroup shared similar COVID-related behaviours, beliefs and attitudes, as well as education and financial comfort. ANOVAs revealed differences between the three subgroups on psychological, political, social and cultural variables. These findings can help to inform effective, tailored approaches for promoting booster uptake that addresses underlying beliefs and concerns about vaccination and remove barriers to vaccination. Our discussion emphasises findings for the Hesitant group as this group may be more responsive to efforts that target vaccine uptake. 

### 4.1. Differences in Demographics and COVID-Related Attitudes

The Hesitant group had a roughly equal proportion of males and females, while the Acceptant group was more than 65% female and the Resistant group more than 60% male. One previous COVID-19 study also found more males in a profile who did not support or comply with the law [35], while a study of booster hesitancy in Australia found females were less likely to have received a booster [21]. In contrast to previous findings [21] and our hypothesis, we did not find any notable differences in education level between the groups. The current study’s sample was relatively well educated. Thus, high education levels may explain the lower hesitancy and resistance rates found among females in this sample. Consistent with other studies, the Hesitant group was slightly younger than the other two groups [21,24,25], supporting our hypothesis about age, although the effect size was small. 

As expected, antivaccination attitudes and lower vaccination uptake were associated with lower compliance and support for other protective measures, such as social distancing and mask wearing. The Hesitant and Resistant groups also reported somewhat greater support for alternative methods of treatment and prevention. Consistent with previous research [25,26], the Hesitant and Resistant groups were less concerned about COVID-19 infection, though these were also small differences. Previous studies specifically examined perceptions of the risk of getting COVID-19, whereas our measure captured concerns about both being infected with COVID-19 and the impact on societal infrastructure and supplies (e.g., the healthcare system being overloaded, groceries and medical supplies running out). As these issues were combined into one scale, differences in perceptions of COVID-19 infection risk may have been obscured. 

To our knowledge, this is the first study on booster uptake to examine perceptions of the impact of the COVID-19 pandemic on political unrest, infringement of civil liberties, damage to the economy and impact on personal finances. The Resistant group showed higher concerns about the impact of the pandemic on political and civil liberties and the economy compared to the Hesitant and Acceptant groups. Interestingly, none of the groups differed in concerns about the impact of the pandemic on their financial circumstances. This finding may also be the result of this sample reporting high levels of education.

Consistent with findings from Malaysia, the Hesitant and Resistant groups showed greater pandemic fatigue than the Acceptant group, with the Resistant group scoring the highest [34]. The relationship between pandemic fatigue and booster uptake has not been extensively studied, so this is a novel finding and supports our hypothesis. 

### 4.2. Motivations behind Willingness to Get Vaccinated

For primary doses, groups did not differ in being motivated by work, social and travel requirements. We found that the Hesitant group were most likely to report external pressures and work, socialising or travel requirements as reasons for getting primary and booster doses. This supports our interpretation of this group as ‘Hesitant’; they might get a vaccine if they feel they ‘have’ to. Conversely, the more strongly antivaccination ‘Resistant’ group were least likely to get a booster for any reason (to protect self and others, if required for freedoms or due to pressure from others). Our findings are largely consistent with previous studies from Spain and the United States demonstrating that the most common motivations for getting primary vaccinations were to protect others and themselves and due to fear of infecting family [91,92], and our largest group (Acceptant) scored highest on this reason. Younger people were also more likely to report getting vaccinated due to social and family pressure, which is consistent with our youngest group, the Hesitant, being most likely to endorse this reason [92]. Our study extends these findings to booster doses, uncovering several extrinsic reasons (e.g., work, travel requirements, etc.) that would motivate the hesitant group to get one. 

### 4.3. Differences in Perceptions of Health Literacy

The Hesitant group scored the lowest on perceptions of health literacy whilst the Resistant group scored the highest. The relatively high score for the Resistant group runs contrary to previous research indicating better health literacy is associated with greater acceptance of COVID-19 vaccines [39,40,41]. Post hoc analyses (see Supplementary Materials, Table S13) indicated that the differences in health literacy between our groups were largely in the ‘critical health literacy’ subscale, which captures tendencies to obtain lots of information about one’s health, thinking about whether health information makes sense, questioning whether information can be trusted, and questioning doctor advice based on own research. With this in mind, the insight provided by our finding is that vaccine-resistant groups and vaccine-acceptant groups share a need to both obtain and question health information while holding very different opinions about vaccination (as discussed above) and using different sources of information (as discussed below). The relatively high education levels of our sample may also explain these unexpected findings.

### 4.4. Differences in COVID-19 Information Gathering

#### 4.4.1. Use of Official and Unofficial Information Sources

All groups reported both consuming and trusting information from casual sources in a similar way. The Acceptant group reported using government/health department websites and checking the news about COVID-19 more frequently than both other groups, consistent with findings from the United Kingdom and Ireland [91]. There were important differences in tendencies to verify the legitimacy of sources, with the Hesitant group reporting that they checked the legitimacy of sources the least, indicating that the Hesitant group might be consuming at least some misinformation and trusting it while not verifying it. These results extend previous findings indicating that booster hesitancy may be associated both with a lack of official information sought and not checking the legitimacy of information that they did consume.

#### 4.4.2. Trust in Information Sources

Community trust in government is reportedly low in Australia and globally, partly attributable to inconsistent policy responses to the pandemic [93,94]. It is also possible that booster hesitancy and resistance might be due to scepticism after vaccines failed to provide the promised benefits that were promised in early claims, such as “the vaccine reduced infections by more than 90%” [95]. With the rapid virus mutation and more available medical data, there has been a need to curb high expectations about vaccination efficacy and set more realistic ones [96], and this was not an easy task for governments to achieve effectively to regain trust in order for the public to accept and process new information.

Policymakers and clinicians are keenly interested in strategies to restore trust to facilitate booster uptake, particularly among at-risk communities. Previous research showed that greater trust in health and government authorities was associated with greater intentions to receive a COVID-19 vaccine (an Australian study [61]) and intentions to receive a booster dose (a Japanese study [27]). While trust in unofficial sources did not differ between groups in this study, levels of trust in official sources did differ in expected ways. The Hesitant group reported being less trusting of science, the media and the government than the Acceptant group and the Resistant group reported the lowest trust across all domains. This is an important finding for policymakers as it provides a target for messaging—it is not just about delivering official COVID-19 information, it is about regaining trust and emphasising the trustworthiness of the information. Amid mixed and changing messages, Basseal et al. stated, ‘timely, clear, and open communication combined with decision making that is evidence informed and as consultative as possible, is essential to maintain population cooperation and trust’ [8] (p. 2). 

### 4.5. The Psychological Profiles of the Vaccine-Hesitant and -Resistant Groups

Statistically significant differences between groups in psychological constructs are discussed in order of their effect sizes [88,89].

The strongest differences (effect size of 0.45) were in psychological reactance, i.e., the dispositional tendency to experience an unpleasant motivational state to resist when one perceives their freedom as being threatened, with the Acceptant group scoring lowest, and the Resistant group scoring highest. According to reactance theory, the level of reactance one experiences is dependent on how large the threat is perceived to be and the individual’s perception of the importance of the freedom being threatened [50]. This suggests that people who resist vaccination place high importance on their individual freedom to make their own decisions and do as they wish; essentially, they do not like being told what to do and will not do it. This association is well documented in pre-COVID research, so dispositional reactance appears to be a key driver of antivaccination attitudes generally [51]. This is also consistent with our finding that the Resistant group may not have been motivated by external pressures or work requirements to get a vaccine dose since they were highly motivated to keep their individual freedom, while the Hesitant group, who were not as high on reactance, were the most likely to get a dose for these external reasons. It is notable that, as the pandemic progressed, the freedom movement grew in Australia, with increasingly large, and in some cases violent, public demonstrations [97].

The second strongest differences (effect size of 0.36) were in amorality, i.e., preference for self-interests and disdain for rules and obligations. Similar to reactance, the Resistant group scored highest, and the Acceptant group lowest. This is consistent with previous research demonstrating an association between pursuing a self-interest and noncompliance with protective public health measures during the early stages of the COVID-19 pandemic [35], as well as with Murphy et al.’s [5] finding that altruism was negatively associated with vaccine hesitancy and resistance.

The next strongest differences were in conspiracy beliefs, with effect sizes of 0.34 for COVID-19 conspiracy beliefs (e.g., the virus was manmade and an attempt to gain control) and 0.30 for general conspiracy mentality (e.g., that secret organisations and powerful groups control the lives of the public). Again, the Hesitant and Resistant groups showed greater conspiracy beliefs than the Acceptant group, and the Resistant group’s conspiracy beliefs were stronger than the Hesitant group’s. The next strongest differences and the last to have a large effect size (0.29) were in need for chaos, i.e., feelings of discontent with current political and social structures [66]. Research from early in the pandemic reported that conspiracy beliefs and a need for chaos formed part of a higher-order mistrust factor that predicted COVID-19 vaccine hesitancy [62]. Our findings extend this research by demonstrating a strong relationship between a need for chaos and booster hesitancy at a later stage of the pandemic. 

Next, with a medium effect size (0.21), were differences in conservativism. The Hesitant and Resistant groups were more socially conservative than the Acceptant group, consistent with prebooster findings on vaccine hesitancy [23,29,60] but contrary to Hagger and Hamilton’s finding that booster intentions were not related to conservatism [23]. There were also medium-sized differences (effect size of 0.18) in cultural tightness, with the Resistant group perceiving greater cultural tightness (i.e., that social norms are strict, and people do not have a great deal of freedom) than both the Hesitant and Acceptant groups, which did not differ from each other. This runs contrary to prevaccine findings whereby those noncompliant with COVID-19 regulations rated their culture as looser [35]. However, it is plausible that the Resistant group perceived a greater lack of freedom as well as intolerance for deviance from social norms than the other groups, despite all participants living in Australia, which is classified as a ‘loose’ culture. The Hesitant and Resistant groups both showed greater persecutory beliefs, such as being suspicious of others’ intentions and thinking others are against them, with a medium effect size (0.15). 

Next, there were medium differences in EAT accuracy (effect size of 0.11), whereby the Hesitant and Resistant groups scored lower on this short measure of verbal reasoning than the Acceptant group but did not differ from each other. Similarly, the Hesitant and Resistant groups also scored lower than the Acceptant group on the personality trait of Agreeableness (ability to put others’ needs above their own), with a medium effect size of 0.10. These findings are consistent with previous studies from earlier stages of the pandemic [5,43,48]. Lastly, there were small but significant differences detected in the personality dimension of Openness (receptivity to new ideas and experiences; effect size of 0.04), with the Hesitant group scoring lower than both other groups, consistent with earlier findings on vaccine hesitancy [43].

Overall, it appears that the strongest and most consistent differences between the groups were rooted in political, cultural and social attitudes and psychological reactance, followed by much weaker differences in some personality dimensions and verbal reasoning. We found no differences in decision-making constructs assessed in this study. Although it is difficult to shift political, cultural and social attitudes and psychological traits, these findings have policy implications for creating tailored information campaigns. These results also provide a critical foundation for the development of psychological theory that extends beyond vaccination intentions.

### 4.6. Implications

**Figure 7:**
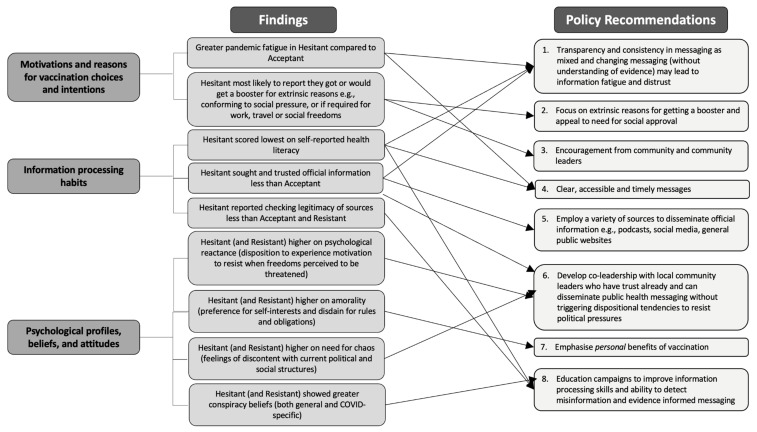
Summary of findings and associated policy recommendations.

This section outlines key results and how they may relate to the evidence-based policy recommendations, as summarised in Figure 7. Nearly one third of the sample fell into the Hesitant group, members of which, overall, reported neither strong beliefs (either positive or negative) about COVID-19 vaccinations and other public health measures or strong willingness or unwillingness to receive a booster dose. The Hesitant group were more likely than the other groups to get a booster if everyone else had if they felt pressure from family, friends or doctors, or if they were required for social, work or travel freedoms, thereby illustrating a key difference from the Resistant group who were not likely to get a booster for any reason. Thus, the Hesitant group is an obvious target for increasing booster uptake and our findings help to better understand their characteristics, attitudes and motives for more effective targeting.

Regarding higher pandemic fatigue in the Hesitant group, coping strategies may be helpful; these could be promoted at a population level with mental health campaigns and taught at an individual level by psychologists, though more research is needed to determine which types of coping strategies are effective in managing and reducing pandemic fatigue. Regarding information consumption, our research suggests there is a critical need to spread accurate information, including information about the reasoning for changes in public health messages, to those who are less likely to actively seek it. Spreading one-size-fits-all information has been shown to be ineffective in producing optimal health outcomes, particularly for at-risk and marginalised communities [98,99,100]. It will therefore be necessary to tailor information to particular communities, keeping in mind that the information should be accessible, appropriate, timely, culturally safe and disseminated in multiple languages. The Hesitant group had the lowest perceptions of health literacy, so it is important to include clear, simple explanations in messaging to this group to help support their understanding and confidence.

In relation to messaging, an important finding from our study is that those less willing to be vaccinated distrust those who commonly deliver public health messages, including governments, scientists and health professionals, which might lead to resistance towards the advice. Thus, there is a need to not only emphasise the trustworthiness of those sources but also to build trust through genuine community engagement. There is a need to disseminate advice through a range of media (which might include a range of social media platforms) and agents or ambassadors (which may include community leaders for subgroups, including religious groups, immigrant communities and popular personalities) which are more trusted by Hesitant groups. Developing co-leadership with local community leaders is an important and effective strategy for facilitating access to, and uptake of, boosters [101]. Codesigning timely health messaging campaigns and health policy, with input from individuals representing diverse communities, is also likely to facilitate their effectiveness.

Misinformation is a major challenge to managing and steering public responses to a pandemic in the digital era. The likeliness of people absorbing misinformation uncritically is an important issue that needs to be addressed, not only for pandemics but for many other issues that involve community response, such as elections. Widespread education efforts are needed to help the general population evaluate the credibility of information from different sources.

Lastly, messaging that emphasises personal benefits (e.g., freedom from being bed-ridden and a potentially serious illness then travelling, fewer chances of dying or getting seriously ill if contracting the virus) may be more effective for tailoring messages to the Hesitant and Resistant than focusing on collective benefits or what is ‘for the greater good’.

### 4.7. Limitations and Future Directions

The findings of this study should be interpreted in consideration of the following limitations. The sample was better educated and on the younger side compared to the Australian general population, with more than half of the sample between 18 and 32 years [102,103,104]. Thus, caution is needed when generalising these findings to older and less educated populations. Participating in the study also required internet access and sufficient literacy levels to be able to complete the survey, so results should be interpreted in lightof potential sampling biases. Additionally, it was beyond the scope of our study to investigate specific populations such as Aboriginal and Torres Strait Islander peoples, immigrants and people living in regional and remote locations who experience unique disparities and circumstances which may affect their vaccination attitudes and uptake. Thus, our findings cannot be generalised to these populations. Future research is needed to better understand how to optimise vaccination uptake in these populations within Australia. Another important limitation was the low number of reported severe side effects in this sample, precluding us from meaningful investigation and conclusions about previous traumatic experiences and booster uptake [55]. Additionally, there may be many reasons for vaccine hesitancy or resistance which were not explored in this study, including previous negative healthcare or vaccination experiences. Future studies must investigate these crucial factors to better understand the uptake of booster doses and other vaccines.

Moreover, this study was cross sectional and observational; thus, no causal links can be determined. Longitudinal research is required to determine predictors of ongoing vaccine choices and the outcomes of vaccination and boosters. For instance, vaccine acceptance was associated with somewhat greater worry about COVID-19 infection, which may be related to consuming more news and information about the virus. Also, information consumption may play a role in forming subjective perceptions of health literacy and risks associated with the virus and vaccines; future research should examine these relationships. While establishing the levels of trust in official and non-official sources of information about COVID-19, our study did not identify the types of personalities and public figures that would be trusted to communicate the required public health messaging. These individuals would likely differ between communities and ground-up, community-led approaches may be needed to identify such figures [101]. Future research could explore this in specific communities to inform how to reach hesitant individuals.

Lastly, while we examined a range of (largely psychological) factors associated with vaccine hesitancy, there are other potentially important factors that were beyond the scope of this study, such as previous traumatic experiences in healthcare, including adverse side effects to vaccinations, and additional sociodemographic factors such as living situation and metrics of socioeconomic status. Different cultural values were also not investigated and may play a role and should be investigated in future research. For example, communitarianism or the value of ‘mate-ship’ in Australia.

## 5. Conclusions

Our study extends the literature on COVID-19 primary vaccine choices and adds to what is known about the drivers of an individual’s decisions around non-mandatory booster doses. Our findings are important as we continue to live with COVID-19 and use booster doses as one of our primary methods of protection. They provide critical insights into the information consumption habits, beliefs, motivations and psychological characteristics associated with a willingness to receive a non-mandated COVID-19 booster dose. The largest group differences in psychological characteristics were found in political, cultural and social attitudes, in particular, reactance, amorality, conspiracy mentality and need for chaos. Understanding the sociodemographic, psychological, social, political and cultural factors that influence vaccine acceptance, hesitance and resistance can help guide and target public health policy. The Hesitant group is an obvious target for increasing booster uptake and an understanding of their characteristics, attitudes, information-sourcing habits/practices and motives can inform more effective and tailored public health communication strategies, thereby facilitating vaccination uptake and compliance with public health protections.

Our findings also have implications for other public health emergencies as they characterise subgroups who may be mistrusting or resistant towards government advice in general. As policies in Australia were similar to those in other countries, these findings have global significance and may inform research and policy relating to COVID-19 and future health threats that require ongoing vaccination, although the application of such lessons may need to be tailored for specific diseases, countries, communities and contexts for optimal efficacy.

## Figures and Tables

**Table 2:** Summary of latent profile analysis results.

Classes	AIC	BIC	SABIC	Entropy	BLRT(p)	LogLik	LogLik(p) *
1	31,438	31,604	31,484	1	-	−15,681	-
2	29,372	29,625	29,441	0.94	0.01	−14,628	<0.001
3	28,697	29,038	28,790	0.91	0.01	−14,271	<0.001
4	28,408	28,836	28,525	0.87	0.01	−14,106	<0.001
5	28,211	28,726	28,351	0.86	0.01	−13,987	<0.001
6	28,129	28,731	28,293	0.84	0.01	−13,926	<0.001
3-class solution							
	Counts and proportions for latent classes			Average probability of class classification accuracy for each class			
	Counts	Proportions		Class 1	Class 2	Class 3	
Class 1	176	0.30		0.95	0.05	0.00	
Class 2	353	0.61		0.04	0.96	0.00	
Class 3	53	0.09		0.03	0.00	0.97	

* *p*-values of the chi-squared test between k and k − 1 solutions.

**Table 3:** ANOVAs and pairwise comparisons between the three profiles in demographics and COVID-19 beliefs and behaviours.

		Mean (SD)			ANOVA			Pairwise Comparisons *p*-Value		
	*α*	Class 1 Hesitant (n = 176)	Class 2 Acceptant (n = 353)	Class 3 Resistant (n = 53)	*F*	*p*-Value	η^2^	c1-2	c1-3	c2-3
		Hesitant	Acceptant	Resistant						
**Demographics**										
Age	-	32.61 (10.9)	35.78 (13.82)	34.15 (10.53)	4.14	<0.05	0.05	0.01	0.62	0.58
**COVID-19 variables: Vaccination and conspiracy beliefs**										
Vaccination Status	-	2.24 (0.63)	2.58 (0.55)	1.38 (1.1)	45.37	<0.001	**0.42**	<0.001	<0.001	<0.001
COVID-19 Conspiracy	0.89	1.42 (0.72)	1.17 (0.45)	2.32 (1.25)	28.94	<0.001	**0.34**	<0.001	<0.001	<0.001
**Reasons for getting primary vaccination doses**										
Regaining Freedom/Work Requirements	0.69	4.23 (1.21)	3.96 (1.44)	4.39 (1.29)	3.47	<0.05	0.07	0.07	0.77	0.16
External Pressures	0.74	2.80 (1.21)	2.09 (1.05)	2.20 (1.06)	21.6	<0.001	**0.33**	<0.001	0.01	0.85
Protecting Self and Others	0.92	5.02 (0.70)	5.75 (0.43)	3.22 (1.17)	149.75	<0.001	**0.79**	<0.001	<0.001	<0.001
**Reasons for getting/would get a booster dose**										
Regaining Freedom/Work Requirements	0.70	4.4 (1.19)	4.03 (1.49)	4.13 (1.51)	4.88	<0.01	0.10	0.005	0.58	0.92
External Pressures	0.75–0.80	2.74 (1.25)	2.18 (1.27)	2.01 (1.04)	13.28	<0.001	0.22	<0.001	0.002	0.65

Note. α = Cronbach’s alpha. η^2^ above 0.25 (bolded) indicates large effect sizes [90,91].

**Table 4:** ANOVAs and pairwise comparisons between the three profiles on information gathering.

		Mean (SD)			ANOVA			Pairwise Comparisons (*p*-Values)		
	*α*	Class 1 (n = 176)	Class 2 (n = 353)	Class 3 (n = 53)	*F*	*p*-Value	η^2^	c1-2	c1-3	c2-3
		Hesitant	Acceptant	Resistant						
Official Sources	0.80	2.42 (0.78)	2.84 (0.79)	2.20 (0.74)	27.64	<0.001	**0.28**	<0.001	0.14	<0.001
Unofficial Sources	0.62	2.49 (0.68)	2.52 (0.66)	2.41 (0.70)	0.56	0.57	0.01	0.89	0.75	0.56
Check News	-	2.95 (1.01)	3.51 (0.86)	2.74 (1.09)	27.64	<0.001	**0.30**	<0.001	0.42	<0.001
Check Source	-	2.98 (1.07)	3.54 (1.12)	3.26 (1.24)	15.37	<0.001	0.18	<0.001	0.30	0.29
Share News	-	2.42 (1.15)	2.81 (1.17)	2.43 (1.29)	7.50	<0.001	0.10	<0.001	1.00	0.12

Note. α = Cronbach’s alpha. η^2^ above 0.25 (bolded) indicates large effect sizes [90,91].

**Table 5:** ANOVAs and pairwise comparisons between the three profiles on psychological factors.

		Mean (SD)			ANOVA			Pairwise Comparisons (*p*-Values)		
	*α*	Class 1 (n = 176)	Class 2 (n = 353)	Class 3 (n = 53)	*F*	*p*-Value	η^2^	c1-2	c1-3	c2-3
		Hesitant	Acceptant	Resistant						
**Psychological Characteristics**										
Agreeableness	0.81	14.55 (3.28)	15.59 (3.16)	14.34 (3.03)	8.25	<0.001	0.10	0.002	0.90	0.02
Conscientiousness	0.68	13.6 (2.81)	14.11 (3.39)	14.26 (2.98)	2.05	0.13	0.03	0.16	0.32	0.94
Extraversion	0.80	9.96 (3.67)	10.28 (3.75)	9.92 (3.59)	0.55	0.58	0.01	0.62	1.00	0.78
Neuroticism	0.77	12.12 (3.33)	12.14 (3.71)	12.4 (3.45)	0.14	0.87	0.00	1.00	0.86	0.87
Openness	0.74	14.51 (3.04)	15.21 (3.37)	15.04 (3.19)	2.96	0.05	0.04	0.04	0.53	0.93
Resilience	0.92	24.19 (7.27)	24.81 (7.51)	24.34 (9.25)	0.44	0.65	0.01	0.63	0.99	0.93
Intolerance	0.91	27.64 (7.08)	27.81 (7.27)	29.19 (8.10)	0.80	0.45	0.01	0.96	0.42	0.48
Reactance	0.90	2.95 (0.60)	2.45 (0.62)	3.23 (0.60)	58.33	<0.001	**0.45**	<0.001	0.01	<0.001
**Cognitive Ability and Decision Making**										
EAT Accuracy	0.72	62.62 (20.01)	69.34 (18.6)	61.45 (22.03)	8.62	<0.001	0.11	<0.001	0.94	0.04
CRT Accuracy	0.67	47.44 (35.26)	50.42 (34.44)	46.7 (37.35)	0.56	0.57	0.01	0.63	0.99	0.77
Bullshit receptivity	0.91	2.38 (0.90)	2.22 (0.87)	2.28 (0.88)	1.77	0.17	0.02	0.14	0.76	0.89
**Political, Cultural and Social Attitudes**										
Conservatism	0.62	3.31 (0.75)	2.92 (0.91)	3.51 (0.86)	19.12	<0.001	0.21	<0.001	0.28	<0.001
Conspiracy mentality	0.83	4.36 (0.83)	3.95 (0.90)	4.89 (0.96)	29.91	<0.001	**0.30**	<0.001	0.001	<0.001
Persecution	0.89	2.42 (0.94)	2.03 (0.83)	2.39 (0.88)	12.53	<0.001	0.15	<0.001	0.98	0.02
Need for Chaos	0.81	2.16 (0.98)	1.65 (0.71)	2.32 (1.15)	25.49	<0.001	**0.29**	<0.001	0.63	<0.001
Cultural Tightness	0.67	4.1 (0.53)	4.16 (0.61)	4.59 (0.58)	15.24	<0.001	0.18	0.48	<0.001	<0.001
Amorality	0.66	2.45 (0.60)	2.11 (0.55)	2.75 (0.61)	38.34	<0.001	**0.36**	<0.001	0.005	<0.001

Note. α = Cronbach’s alpha. η^2^ above 0.25 (bolded) indicates large effect sizes [90,91].

## Data Availability

The data presented in this study are openly available in the OSF data repository at https://doi.org/10.17605/OSF.IO/UV2Q7.

## Supplementary Materials

The following supporting information can be downloaded at: https://www.mdpi.com/article/10.3390/vaccines11050907/s1, Table S1: Summary of COVID-19 restrictions during data collection period: 29 January to 11 March 2022; Table S2: Exploratory factor analysis of COVID-19 beliefs and Vaccination Attitudes Examination Scale (N = 582); Table S3: Exploratory factor analysis of the Multidimensional COVID-19 Worry Index (N = 582); Table S4: Exploratory factor analysis of COVID-19 readiness (N = 582); Table S5: Exploratory factor analysis of compliance attitudes (current) items (N = 582); Table S6: Exploratory factor analysis of compliance attitudes (before December 2021) items (N = 582); Table S7: Exploratory factor analysis of trust items (N = 582); Table S8: Exploratory factor analysis of vaccination reasons (N = 554); Table S9: Exploratory factor analysis of booster reasons (N = 554); Table S10: Bogus items; Table S11: Logic checks; Table S12: Frequencies for number of flags received by participants in total; Table S13: Sample characteristics (N = 582); Table S14: ANOVAs and pairwise comparisons between the three profiles in health literacy subscale.

## References

[1] World Health Organization Global COVID-19 Vaccination Strategy in a Changing World. https://www.who.int/publications/m/item/global-covid-19-vaccination-strategy-in-a-changing-world--july-2022-update.

[2] World Health Organization WHO Coronavirus (COVID-19) Dashboard. https://covid19.who.int/.

[3] Bennett N.G., Bloom D.E., Ferranna M. (2022). Factors underlying COVID-19 vaccine and booster hesitancy and refusal, and incentivizing vaccine adoption. PLoS ONE.

[4] Cristea D., Ilie D.-G., Constantinescu C., Fîrțală V. (2022). Acceptance, Hesitancy, and Refusal in Anti-COVID-19 Vaccination: A Cluster Analysis Aiming at the Typology behind These Three Concepts. Vaccines.

[5] Murphy J., Vallières F., Bentall R.P., Shevlin M., McBride O., Hartman T.K., McKay R., Bennett K., Mason L., Gibson-Miller J. (2021). Psychological characteristics associated with COVID-19 vaccine hesitancy and resistance in Ireland and the United Kingdom. Nat. Commun..

[6] Moffitt T.E., Caspi A., Ambler A., Bourassa K., Harrington H., Hogan S., Houts R., Ramrakha S., Wood S.L., Poulton R. (2022). Deep-seated psychological histories of COVID-19 vaccine hesitance and resistance. PNAS Nexus.

[7] Singh P., Dhalaria P., Kashyap S., Soni G.K., Nandi P., Ghosh S., Mohapatra M.K., Rastogi A., Prakash D. (2022). Strategies to overcome vaccine hesitancy: A systematic review. Syst. Rev..

[8] Basseal J.M., Bennett C.M., Collignon P., Currie B.J., Durrheim D.N., Leask J., McBryde E.S., McIntyre P., Russell F.M., Smith D.W. (2022). Key lessons from the COVID-19 public health response in Australia. Lancet Reg. Health—West. Pac..

[9] Mathieu E., Ritchie H., Rodes-Guirao L., Appel C., Gavrilov D., Giattino C., Hasell J., Macdonald B., Dattani S., Beltekian D. (2020). Coronavirus (COVID-19) Deaths. https://ourworldindata.org/covid-deaths.

[10] Australian Government Department of Health COVID-19 Vaccine Rollout Update—30 June 2021. https://www.health.gov.au/resources/publications/covid-19-vaccine-rollout-update-30-june-2021?language=en.

[11] Nicholas J. How Australia’s Coronavirus Vaccine Rollout Really Compares with Other Countries. https://www.theguardian.com/news/datablog/2021/apr/2014/how-australias-coronavirus-vaccine-rollout-really-compares-with-other-countries.

[12] Australian Government Department of Health COVID-19 Vaccine Rollout Update—17 December 2021. https://www.health.gov.au/resources/publications/covid-19-vaccine-rollout-update-17-december-2021.

[13] Australian Government Department of Health (2022). Coronavirus (COVID-19) at a Glance. https://www.health.gov.au/resources/publications/coronavirus-covid-19-at-a-glance-10-january-2022?language=en.

[14] Timms P., Lloyd M. COVID-19 Testing under Pressure across Australia, as Rapid Antigen Tests Remain Hard to Find Amid Long Delays for PCRs. https://www.abc.net.au/news/2022-01-05/covid-testing-pcr-delays-rat-test-supply-issues/100738982.

[15] Talbot J. We Are Not Going Back to Shutting Down Peoples’ Lives’ Says the Prime Minister amid Record COVID-19 Cases across Australia. https://www.skynews.com.au/australia-news/coronavirus/we-are-not-going-back-to-shutting-down-peoples-lives-says-the-prime-minister-amid-record-covid19-cases-across-australia/news-story/16c17b55522e55582b55512df047283b047289b047283a047286c047286f.

[16] Australian Medical Association (2021). Australia Must Move Quickly to Speed Up COVID-19 Booster Program. https://www.ama.com.au/articles/australia-must-move-quickly-speed-covid-19-booster-program.

[17] Liu B., Gidding H., Stepien S., Cretikos M., Macartney K. (2022). Relative effectiveness of COVID-19 vaccination with 3 compared to 2 doses against SARS-CoV-2 B.1.1.529 (Omicron) among an Australian population with low prior rates of SARS-CoV-2 infection. Lancet.

[18] Office for National Statistics Self-Reported Long COVID after Infection with the Omicron Variant in the UK: 6 May 2022. https://www.ons.gov.uk/peoplepopulationandcommunity/healthandsocialcare/conditionsanddiseases/bulletins/selfreportedlongcovidafterinfectionwiththeomicronvariant/6may2022.

[19] Shen S.C., Dubey V. (2019). Addressing vaccine hesitancy: Clinical guidance for primary care physicians working with parents. Can. Fam. Physician.

[20] Leask J. (2011). Target the fence-sitters. Nature.

[21] Biddle N., Sollis K. (2022). Who Wants to Get Boosted? COVID-19 Vaccine Uptake in Australia in January 2022.

[22] Nicholas J. Australia Won’t Reach 80% Covid Booster Rate until Well into 2022, Analysis Shows. https://www.theguardian.com/australia-news/2021/dec/2019/australia-wont-reach-2080-covid-booster-rate-until-well-into-2022-analysis-shows.

[23] Hagger M.S., Hamilton K. (2022). Predicting COVID-19 booster vaccine intentions. Appl. Psychol. Health Well-Being.

[24] Klugar M., Riad A., Mohanan L., Pokorná A. (2021). COVID-19 Vaccine Booster Hesitancy (VBH) of Healthcare Workers in Czechia: National Cross-Sectional Study. Vaccines.

[25] Paul E., Fancourt D. (2022). Predictors of uncertainty and unwillingness to receive the COVID-19 booster vaccine: An observational study of 22,139 fully vaccinated adults in the UK. Lancet Reg. Health Eur..

[26] Tan K.Y.K., Soh A.S.E., Ong B.W.L., Chen M.I., Griva K. (2022). Determining the Prevalence and Correlates of COVID-19 Booster Vaccine Hesitancy in the Singapore Population Following the Completion of the Primary Vaccination Series. Vaccines.

[27] Yadete T., Batra K., Netski D.M., Antonio S., Patros M.J., Bester J.C. (2021). Assessing Acceptability of COVID-19 Vaccine Booster Dose among Adult Americans: A Cross-Sectional Study. Vaccines.

[28] Yoshida M., Kobashi Y., Kawamura T., Shimazu Y., Nishikawa Y., Omata F., Zhao T., Yamamoto C., Kaneko Y., Nakayama A. (2022). Factors Associated with COVID-19 Vaccine Booster Hesitancy: A Retrospective Cohort Study, Fukushima Vaccination Community Survey. Vaccines.

[29] Roberts H.A., Clark D.A., Kalina C., Sherman C., Brislin S., Heitzeg M.M., Hicks B.M. (2022). To vax or not to vax: Predictors of anti-vax attitudes and COVID-19 vaccine hesitancy prior to widespread vaccine availability. PLoS ONE.

[30] Lazarus J.V., Ratzan S.C., Palayew A., Gostin L.O., Larson H.J., Rabin K., Kimball S., El-Mohandes A. (2021). A global survey of potential acceptance of a COVID-19 vaccine. Nat. Med..

[31] World Health Organization Pandemic Fatigue: Reinvigorating the Public to Prevent COVID-19. https://apps.who.int/iris/handle/10665/335820.

[32] Lilleholt L., Zettler I., Betsch C., Böhm R. (2020). Pandemic fatigue: Measurement, correlates, and consequences. PsyArXiv.

[33] Bodas M., Kaim A., Velan B., Ziv A., Jaffe E., Adini B. (2022). Overcoming the effect of pandemic fatigue on vaccine hesitancy-Will belief in science triumph?. J. Nurs. Sch..

[34] Wong L.P., Alias H., Siaw Y.-L., Muslimin M., Lai L.L., Lin Y., Hu Z. (2022). Intention to receive a COVID-19 vaccine booster dose and associated factors in Malaysia. Hum. Vaccines Immunother..

[35] Kleitman S., Fullerton D.J., Zhang L.M., Blanchard M.D., Lee J., Stankov L., Thompson V. (2021). To comply or not comply? A latent profile analysis of behaviours and attitudes during the COVID-19 pandemic. PLoS ONE.

[36] Loomba S., de Figueiredo A., Piatek S.J., de Graaf K., Larson H.J. (2021). Measuring the impact of COVID-19 vaccine misinformation on vaccination intent in the UK and USA. Nat. Hum. Behav..

[37] Allen K., Lambert S.B., Yuen A., Pourmarzi D. (2022). Factors associated with COVID-19 booster vaccine willingness among migrants from the Eastern Mediterranean living in Australia: A cross-sectional study. Res. Sq..

[38] Ratzan S.C., Parker R.M., Seldon C.R., Zorn M., Ratzan S.C., Parker R.M. (2000). Introduction. National Library of Medicine Current Bibliographies in Medicine: Health Literacy.

[39] Kricorian K., Civen R., Equils O. (2022). COVID-19 vaccine hesitancy: Misinformation and perceptions of vaccine safety. Hum. Vaccines Immunother..

[40] Montagni I., Ouazzani-Touhami K., Mebarki A., Texier N., Schück S., Tzourio C. (2021). Acceptance of a COVID-19 vaccine is associated with ability to detect fake news and health literacy. J. Public Health.

[41] Zhang H., Li Y., Peng S., Jiang Y., Jin H., Zhang F. (2022). The effect of health literacy on COVID-19 vaccine hesitancy among community population in China: The moderating role of stress. Vaccine.

[42] Pennycook G., Cheyne J.A., Barr N., Koehler D.J., Fugelsang J.A. (2015). On the reception and detection of pseudo-profound bullshit. Judgm. Decis.-Mak..

[43] Roshchina Y., Roshchin S., Rozhkova K. (2022). Determinants of COVID-19 vaccine hesitancy and resistance in Russia. Vaccine.

[44] Salerno L., Craxì L., Amodio E., Lo Coco G. (2021). Factors Affecting Hesitancy to mRNA and Viral Vector COVID-19 Vaccines among College Students in Italy. Vaccines.

[45] Frederick S. (2005). Cognitive Reflection and Decision Making. J. Econ. Perspect..

[46] Sinayev A., Peters E. (2015). Cognitive reflection vs. calculation in decision making. Front. Psychol..

[47] Campitelli G., Gerrans P. (2014). Does the cognitive reflection test measure cognitive reflection? A mathematical modeling approach. Mem. Cogn..

[48] Halstead I.N., McKay R.T., Lewis G.J. (2022). COVID-19 and seasonal flu vaccination hesitancy: Links to personality and general intelligence in a large, UK cohort. Vaccine.

[49] Cattell R.B. (1963). Theory of fluid and crystallized intelligence: A critical experiment. J. Educ. Psychol..

[50] Steindl C., Jonas E., Sittenthaler S., Traut-Mattausch E., Greenberg J. (2015). Understanding Psychological Reactance: New Developments and Findings. Z. Psychol..

[51] Hornsey M.J., Harris E.A., Fielding K.S. (2018). The psychological roots of anti-vaccination attitudes: A 24-nation investigation. Health Psychol..

[52] Sprengholz P., Betsch C., Böhm R. (2021). Reactance revisited: Consequences of mandatory and scarce vaccination in the case of COVID-19. Appl. Psychol. Health Well-Being.

[53] Sprengholz P., Felgendreff L., Böhm R., Betsch C. (2022). Vaccination policy reactance: Predictors, consequences, and countermeasures. J. Health Psychol..

[54] Albarracin D., Jung H., Song W., Tan A., Fishman J. (2021). Rather than inducing psychological reactance, requiring vaccination strengthens intentions to vaccinate in US populations. Sci. Rep..

[55] Christou-Ergos M., Wiley K.E., Leask J. (2023). Willingness to receive a vaccine is influenced by adverse events following immunisation experienced by others. Vaccine.

[56] Carleton R.N., Norton M.A., Asmundson G.J. (2007). Fearing the unknown: A short version of the Intolerance of Uncertainty Scale. J. Anxiety Disord..

[57] McNeil A., Purdon C. (2022). Anxiety disorders, COVID-19 fear, and vaccine hesitancy. J. Anxiety Disord..

[58] Zheng Y.-B., Sun J., Liu L., Zhao Y.-M., Yan W., Yuan K., Su S.-Z., Lu Z.-A., Huang Y.-T., Liu L. (2021). COVID-19 Vaccine-Related Psychological Stress Among General Public in China. Front. Psychiatry.

[59] Galanis P., Katsiroumpa A., Sourtzi P., Siskou O., Konstantakopoulou O., Katsoulas T., Kaitelidou D. (2023). COVID-19-Related Burnout and Intention of Fully Vaccinated Individuals to Get a Booster Dose: The Mediating Role of Resilience. Vaccines.

[60] Peng Y. (2022). Politics of COVID-19 vaccine mandates: Left/right-wing authoritarianism, social dominance orientation, and libertarianism. Personal. Individ. Dif..

[61] Edwards B., Biddle N., Gray M., Sollis K. (2021). COVID-19 vaccine hesitancy and resistance: Correlates in a nationally representative longitudinal survey of the Australian population. PLoS ONE.

[62] Freeman D., Loe B.S., Chadwick A., Vaccari C., Waite F., Rosebrock L., Jenner L., Petit A., Lewandowsky S., Vanderslott S. (2020). COVID-19 vaccine hesitancy in the UK: The Oxford coronavirus explanations, attitudes, and narratives survey (Oceans) II. Psychol. Med..

[63] Nazlı Ş.B., Yığman F., Sevindik M., Deniz Özturan D. (2022). Psychological factors affecting COVID-19 vaccine hesitancy. Ir. J. Med. Sci..

[64] Gelfand M.J., Raver J.L., Nishii L., Leslie L.M., Lun J., Lim B.C., Duan L., Almaliach A., Ang S., Arnadottir J. (2011). Differences Between Tight and Loose Cultures: A 33-Nation Study. Science.

[65] Ng J.H., Tan E.K. (2021). COVID-19 vaccination and cultural tightness. Psychol. Med..

[66] Mathieu E., Ritchie H., Rodés-Guirao L., Appel C., Giattino C., Hasell J., Macdonald B., Dattani S., Beltekian D., Ortiz-Ospina E. (2020). Coronavirus Pandemic (COVID-19). https://ourworldindata.org/coronavirus.

[67] Kleitman S., Fullerton D.J., Zhang L.M., Aidman E. (2022). The Role of Cognitive Fitness Constructs in Predicting Mental Well-Being and Its Recovery after the First COVID-19 Lockdown in Australia.

[68] Martin L.R., Petrie K.J. (2017). Understanding the dimensions of anti-vaccination attitudes: The Vaccination Attitudes Examination (VAX) Scale. Ann. Behav. Med..

[69] Cuadrado E., Maldonado M.A., Tabernero C., Arenas A., Castillo-Mayén R., Luque B. (2021). Construction and Validation of a Brief Pandemic Fatigue Scale in the Context of the Coronavirus-19 Public Health Crisis. Int. J. Public Health.

[70] Chinn D., McCarthy C. (2013). All Aspects of Health Literacy Scale (AAHLS): Developing a tool to measure functional, communicative and critical health literacy in primary healthcare settings. Patient Educ. Couns..

[71] Donnellan M.B., Oswald F.L., Baird B.M., Lucas R.E. (2006). The Mini-IPIP Scales: Tiny-yet-effective measures of the Big Five factors of personality. Psychol. Assess..

[72] Stankov L. (1997). Gf-Gc Quickie Test Battery.

[73] Jackson S.A., Kleitman S. (2014). Individual differences in decision-making and confidence: Capturing decision tendencies in a fictitious medical test. Metacogn. Learn..

[74] Jackson S.A., Kleitman S., Howie P., Stankov L. (2016). Cognitive abilities, monitoring confidence, and control thresholds explain individual differences in heuristics and biases. Front. Psychol..

[75] Toplak M.E., West R.F., Stanovich K.E. (2014). Assessing miserly information processing: An expansion of the Cognitive Reflection Test. Think. Reason..

[76] Campbell-Sills L., Stein M.B. (2007). Psychometric analysis and refinement of the Connor-davidson Resilience Scale (CD-RISC): Validation of a 10-item measure of resilience. J. Trauma. Stress.

[77] Hong S.-M., Page S. (1989). A psychological reactance scale: Development, factor structure and reliability. Psychol. Rep..

[78] Shen L., Dillard J.P. (2005). Psychometric properties of the Hong Psychological Reactance Scale. J. Personal. Assess..

[79] Everett J.A.C. (2013). The 12 item Social and Economic Conservatism Scale (SECS). PLoS ONE.

[80] Bruder M., Haffke P., Neave N., Nouripanah N., Imhoff R. (2013). Measuring individual differences in generic beliefs in conspiracy theories across cultures: Conspiracy mentality questionnaire. Front. Psychol..

[81] Melo S., Corcoran R., Shryane N., Bentall R.P. (2009). The Persecution and Deservedness Scale. Psychol. Psychother. Theory Res. Pract..

[82] Petersen M., Osmunden M., Areceneaux K. (2023). The “need for chaos” and motivations to share hostile political rumors. Am. Political Sci. Rev..

[83] Stankov L., Knezevic G. (2005). Amoral social attitudes and value systems among Serbs and Australians. Aust. J. Psychol..

[84] Hagenaars J.A., McCutcheon A.L. (2002). Applied Latent Class Analysis.

[85] Marsh H.W., Lüdtke O., Trautwein U., Morin A.J.S. (2009). Classical Latent Profile Analysis of Academic Self-Concept Dimensions: Synergy of Person- and Variable-Centered Approaches to Theoretical Models of Self-Concept. Struct. Equ. Model. Multidiscip. J..

[86] Spurk D., Hirschi A., Wang M., Valero D., Kauffeld S. (2020). Latent profile analysis: A review and “how to” guide of its application within vocational behavior research. J. Vocat. Behav..

[87] Sauder D., DeMars C. (2019). An Updated Recommendation for Multiple Comparisons. Adv. Methods Pract. Psychol. Sci..

[88] Cohen J. (1988). Statistical Power Analysis for the Behavioural Sciences.

[89] Tabachnick B.G., Fidell L.S. (2013). Using Multivariate Statistics.

[90] R Core Team (2021). R: A Language and Environment for Statistical Computing.

[91] Moore R., Purvis R.S., Hallgren E., Willis D.E., Hall S., Reece S., CarlLee S., Judkins H., McElfish P.A. (2022). Motivations to Vaccinate Among Hesitant Adopters of the COVID-19 Vaccine. J. Community Health.

[92] Sauch Valmaña G., Fuster-Casanovas A., Ramírez-Morros A., Rodoreda Pallàs B., Vidal-Alaball J., Ruiz-Comellas A., Miró Catalina Q. (2022). Motivation for Vaccination against COVID-19 in Persons Aged between 18 and 60 Years at a Population-Based Vaccination Site in Manresa (Spain). Vaccines.

[93] Monash University The Australian Government is Trapped in a Cycle of Distrust—How Can It Break Out?. https://lens.monash.edu/@politics-society/2022/2005/2002/1384632/the-australian-government-is-trapped-in-a-cycle-of-distrust-how-can-it-break-out.

[94] Edelman Trust Barometer 2022 Australia. https://edelman.com.au/trust-barometer-2022-australia.

[95] Garde D., Herper M. Pfizer and BioNTech to Submit COVID-19 Vaccine Data to FDA as Full Results Show 95% Efficacy. https://www.statnews.com/2020/2011/2018/pfizer-biontech-covid2019-vaccine-fda-data/.

[96] Branswell H. COVID-19 Vaccines Never Promised Perfection. Experts Say It’s Time to Curb Our Highest Expectations. https://www.pbs.org/newshour/health/covid-19-vaccines-never-promised-perfection-experts-say-its-time-to-curb-our-highest-expectations.

[97] Van der Zwet K., Barros A.I., van Engers T.M., Sloot P.M.A. (2022). Emergence of protests during the COVID-19 pandemic: Quantitative models to explore the contributions of societal conditions. Humanit. Soc. Sci. Commun..

[98] Kreuter M.W., Bull F.C., Clark E.M., Oswald D.L. (1999). Understanding how people process health information: A comparison of tailored and nontailored weight-loss materials. Health Psychol..

[99] Fernandez S., Wagner E., Hospital M., Howard M., Morris S. (2019). Social media based strategies to reach Hispanic young adults with tailored sexual health information. Soc. Work Soc. Sci. Rev..

[100] Kreps G.L., Sparks L. (2008). Meeting the health literacy needs of immigrant populations. Patient Educ. Couns..

[101] Desborough J., Wright M., Parkinson A., Hall Dykgraaf S., Ball L., Dut G.M., Sturgiss E., de Toca L., Kidd M. (2022). What strategies have been effective in optimising COVID-19 vaccine uptake in Australia and internationally?. Aust. J. Gen. Pract..

[102] Australian Bureau of Statistics National, State and Territory Population. https://abs.gov.au/statistics/people/population/national-state-and-territory-population/latest-release.

[103] Australian Bureau of Statistics Regional Population by Age and Sex. https://abs.gov.au/statistics/people/population/regional-population-age-and-sex/2021.

[104] Australian Bureau of Statistics Education and Work, Australia. https://www.abs.gov.au/statistics/people/education/education-and-work-australia/may-2022.

