# Supplementary material for: The Psychology of COVID-19 Booster Hesitancy, Acceptance and Resistance in Australia
Source: Vaccines (Basel). 2023 Apr 27;11(5):907. doi: 10.3390/vaccines11050907 (PMC10222735; doi:10.3390/vaccines11050907)
Supplement: Supplementary file 1 [file vaccines-11-00907-s001.zip › vaccines-2316806-supplementary.pdf]

Supplementary Material

"The Psychology of COVID-19 Booster Hesitancy, Acceptance and Resistance"

'Vaccines'

Special Issue "The Willingness Towards Vaccination: A Focus on Non-Mandatory Vaccinations"

Sabina Kleitman[1*], Dayna J. Fullerton[1], Marvin K. H. Law[1], Matthew D. Blanchard[1], Rachel Campbell[1], Margaret-Ann Tait[1,2], Jennifer Schulz[3,4,5], Jihyun Lee[6], Lazar Stankov[1] and Madeleine T. King[1]

1 School of Psychology, Faculty of Science, University of Sydney, Sydney, NSW 2006, Australia; dayna.fullerton@sydney.edu.au (D.J.F.); marvin.law@sydney.edu.au (M.K.H.L.); matthew.blanchard@sydney.edu.au (M.D.B.); r.campbell@sydney.edu.au (R.C.); margaret-ann.tait@sydney.edu.au (M.-A.T.); lazar.stankov@sydney.edu.au (L.S.); madeleine.king@sydney.edu.au (M.T.K.)
2 Sydney Nursing School, Faculty of Medicine and Health, University of Sydney, Sydney, NSW 2006, Australia
3 Faculty of Law and Justice, University of New South Wales, Sydney, NSW 2052, Australia
4 Faculty of Medicine and Health, University of New South Wales, Sydney, NSW 2052, Australia; jennifer.moore@unsw.edu.au
5 School of Public Health, Faculty of Health and Environmental Sciences, Auckland University of Technology, Auckland 0627, New Zealand
6 School of Education, Faculty of Arts, Design and Architecture, University of New South Wales, Sydney, NSW 2052, Australia; jihyun.lee@unsw.edu.au

*Correspondence: sabina.kleitman@sydney.edu.au; Tel.: +61 2 9351 7703

Table S1. Summary of COVID-19 restrictions during data collection period: 29 January to 11 March 2022

	VIC	NSW	QLD	TAS	WA	SA	NT	ACT
**Face masks**	From 23 Dec:<br>Mandatory indoors and at large outdoor events.<br><br>From 25 Feb:<br>Only required on public transport; in hospitals and care facilities; for workers in hospitality, retail, and the court system; students in year 3 or above; childcare and primary school workers; workers at indoor events with more than 30,000 people.	From 24 Dec:<br>Mandatory indoors and strongly encouraged where cannot maintain social distancing.<br><br>From 25 Feb:<br>Only mandatory on public transport, planes, and indoors at airports, hospitals, aged and disability care facilities, corrections facilities and indoor music festivals with >1000 people.<br><br>Encouraged for indoor settings where you cannot maintain a safe distance from others.	From 2 Jan:<br>Mandatory indoors.<br><br>From 4 March:<br>Only required on public transport, in airports, on planes, in prisons, and in disability, hospital, and aged care settings.<br><br>Recommended whenever you can't socially distance.	From 21 Dec:<br>Mandatory indoors.<br><br>From 5 March:<br>Masks no longer required in retail settings.<br><br>From March 11:<br>No longer required in hospitality venues and offices.	From 23 Dec:<br>Mandatory indoors.<br><br>From 7 Jan:<br>Only required in hospitals, aged and disability care, public transport and rideshare.<br><br>From 16 Jan:<br>Mandatory in all public indoor spaces in the Perth and Peel regions.<br><br>From 27 Jan:<br>Mandatory indoors for Wheatbelt and Great Southern regions.<br><br>From 21 Feb:<br>Masks mandate for all indoor public settings expanded state-wide.	Mandatory in indoor public spaces.	From 31 Dec:<br>Mandatory indoors.<br><br>From 29 Jan:<br>Mandatory outdoors.<br><br>5 Feb:<br>Outdoor mask mandated lifted.<br><br>7 March:<br>Only required in health, aged and disability care facilities, prisons, and airports.<br><br>Indoor mask mandate still in place in Big Rivers region.	From 22 Dec:<br>Mandatory indoors.<br><br>From 25 Feb:<br>Only mandatory on public transport, in hospitals and aged-care facilities, for teachers and childhood educators, for students in years 7 or 12.
**Isolation and testing**	From 12 Jan:	From 31 Dec:		From 26 Feb:			Vaccinated COVID-19 cases	

	COVID-19 cases must self-isolate for 7 days or until they get a negative result. Close contacts must isolate for 7 days. Exposed persons must get tested.	COVID-19 cases must self-isolate for 7 days. If still symptomatic on day 7, they must remain in isolation until they get a negative result. Close contracts must get tested and isolate and may leave isolation on day 7 if they get a negative RAT on day 6.		Household contacts must isolate for 7 days from date of diagnosis of household case			must isolate for 7 days. Unvaccinated must isolate for 10 days.	
**Proof of vaccination**	Required for hair and beauty services, restaurants, cafes, bars, pubs		Required for hospitality and tourism venues				Required for pubs, clubs, casinos, restaurants, cinemas, ticketed events with 500+ people in urban centres and 100+ in non-urban centres	
**Check-in**	<u>From 18 Feb:</u><br>No longer required at retail venues, schools, and many workplaces. Still required in hospitality and entertainment venues	<u>From 27 Dec:</u><br>Compulsory in hospitality and retail shops<br><br><u>From 18 Feb:</u><br>Only required in certain settings including nightclubs or music festivals with 1000+ people.					Required everywhere<br><br><u>From 2 March:</u><br>Only required in pubs, bars clubs, restaurants, cinemas/theatres, casinos, ticketed events	<u>From 11 Feb:</u><br>Only required at bars, pubs, clubs

**Hospitality venues**	One person per 2 square metres.<br><br><u>From 18 Feb:</u><br>No density limits. Dancefloors re-opened.	<u>From 27 Dec:</u><br>One person per 2 square metres indoors.<br><br>Singing and dancing not permitted.<br><br><u>From 18 Feb:</u><br>No capacity limits. Singing and dancing permitted.		Check-in required.<br><br><u>From 20 Feb:</u><br>Check-in only required in bars, pubs, clubs, hotels		50% capacity limit		<u>From 8 Jan:</u><br>Patrons must be seated. No dancing.
**Interstate travel**	No restrictions	Unvaccinated close contacts may not enter. Fully vaccinated close contacts can enter but need to self-isolate for 7 days.<br><br>Residents who are close contacts interstate can return to self-isolate.	Entry pass required for all travellers<br><br>Those required to isolate due to contact with a COVID case in the past 14 days cannot enter.<br><br><u>1 Jan:</u><br>Arrivals from hotspots can enter with a negative RAT	Fully vaccinated travellers allowed to enter without quarantine<br><br><u>From 26 Feb:</u><br>No restrictions for travellers to Tasmania regardless of vaccination status	No visitors from other states or territories permitted.	Travellers from NSW, ACT, VIC need a negative test to enter<br><br>Unvaccinated require an exemption	Arrivals required to take a RAT within two hours of arrival, on Day 3, and Day 6<br><br><u>From 1 Feb:</u><br>Testing no longer required for vaccinated arrivals. Unvaccinated unable to enter without exemption.<br><br><u>From 18 Feb:</u><br>No requirements for arrivals	No restrictions except for close contacts who are required to get an exemption and self-isolate

Sources

VIC

- https://www.theage.com.au/national/victoria/mandatory-masks-indoors-for-victorians-from-tonight-20211223-p59jsn.html
- https://business.vic.gov.au/news-and-updates/2022/return-to-the-office-mask-rules-and-vaccination-requirement-updates
- https://www.premier.vic.gov.au/density-limit-dancefloor-qr-code-restrictions-ease

NSW

- https://www.nsw.gov.au/media-releases/covid-19-settings-in-place
- https://www.9news.com.au/national/coronavirus-update-nsw-restrictions-ease-on-mask-wearing-work-from-home-directives-dancing-in-nightclubs-allowed/150c2f73-3533-41bd-a7b1-63e2c53e597b
- https://aapi.org.au/Web/News/Articles/NSWCOVID23Dec.aspx
- https://www.nsw.gov.au/media-releases/nsw-updates-covid-settings-and-makes-rapid-antigen-tests-free
- https://www.nsw.gov.au/media-releases/covid-19-settings-in-place
- https://www.carersnsw.org.au/news/covid-19-update-for-carers-in-nsw-07-01

QLD

- https://www.mbqld.com.au/news-and-publications/news/covid-19-update-masks-from-2-january
- https://www.abc.net.au/news/2022-03-04/qld-coronavirus-covid19-mask-rules-density-restrictions-ease/100860862

TAS

- https://www.ccc.tas.gov.au/living/environment/covid-19-support-and-information/#:~:text=In%20accordance%20with%20public%20health,Indoor%20workplaces%20such%20as%20offices
- https://www.abc.net.au/news/2022-03-04/tas-covid-masks-new-rules/100881994

WA

- https://unitingwa.org.au/news-and-resources/media-releases/covid-19-coronavirus-updates/

SA

- https://www.abc.net.au/news/2022-02-25/covid-mask-rules-australian-states-territories/100854564

NT

- https://www.abc.net.au/news/2022-02-25/covid-mask-rules-australian-states-territories/100854564
- https://www.abc.net.au/news/2022-03-02/mask-mandate-northern-territory-covid/100873020
- https://createsend.com/t/t-6ED4E7C79CFBB66B2540EF23F30FEDED
- https://mediareleases.nt.gov.au/

ACT

- https://www.covid19.act.gov.au/news-articles/act-reintroduces-indoor-mask-wearing-and-aged-care-visitor-restrictions
- https://www.abc.net.au/news/2022-02-25/covid-mask-rules-australian-states-territories/100854564#act

Original measures

COVID-Related Beliefs

For each statement, please choose the option that best represents your opinion.
1=*strongly disagree*; 5=*strongly agree*

1. Social distancing is effective in slowing the spread of COVID-19
2. Wearing masks for children is unnecessary and adds burden on children and parents
3. Testing, tracking, and tracing every potential case of COVID-19 are effective measures to minimize the spread
4. Ventilation in indoor areas (e.g., school, work, entertainment venues) is effective in slowing the spread of COVID-19
5. Taking a particular type of supplement/vitamin is effective in preventing getting infected by COVID-19
6. Mandatory COVID-19 vaccination requirements violate my civil rights
7. Social distancing practices should remain in use until the COVID-19 pandemic is under control
8. We should rely on people getting COVID-19 to build up (herd) immunity
9. People should use QR codes when required
10. Keeping enclosed spaces well-ventilated should be mandatory for schools, businesses, and workplaces
11. Oral and nasal hygiene (flossing and saline wash) should be recommended COVID-19 prevention measures
12. Discrimination law should protect everyone at their jobs who chooses not to get vaccinated (whether or not they have medical reasons preventing them from doing so)
13. Lockdown policies are essential to keep in place until the COVID-19 pandemic is under control
14. Immunization of children under 16 years of age is unnecessary and problematic
15. Wearing face masks in public places is effective in slowing the spread of COVID-19
16. Self-testing for COVID-19 by doing my own swab using TGA approved rapid test kits is effective in slowing the spread of the pandemic
17. Taking a particular type of supplement/vitamin assists in recovering quickly if infected by COVID-19
18. Social distancing and lockdown policies are detrimental to our economy
19. Anti-vaccination views should be a protected attribute under the Equal Opportunity Act (law to ensure employees are not unfairly disadvantaged)
20. Inhaling steam e.g., with salts/oils should be used to prevent and to treat the COVID-19 infection
21. Lockdowns are effective in slowing the spread of COVID-19
22. COVID-19 Vaccines are effective in slowing the spread of the pandemic
23. Continuing hand hygiene is important in controlling the pandemic spread
24. Alternative medicine like consuming certain teas, herbs, minerals, or supplements are good preventative measures for COVID-19

Multidimensional COVID-19 Worry Index

To what extent do the following statements describe how you feel amid the COVID-19 pandemic?
1=never; 4=always

1. I am nervous when I think about the pandemic
2. I am anxious about losing money due to COVID-19
3. I am concerned about the possibility of another lockdown in the city/region where I live
4. I am nervous about grocery stores running out of food and/or other supplies
5. I am worried about my health due to COVID-19
6. I am nervous about political systems failing

7. I am concerned about taking public transport due to COVID-19
8. I am worried about becoming unemployed due to the pandemic
9. I am stressed about our healthcare system being overloaded
10. I am stressed about leaving my house
11. I am concerned about the health of my family members due to COVID-19
12. I am worried about political unrest in Australia
13. I am worried about pharmacies running out of medicines/essential health supplies
14. I am worried about someone I love dying due to COVID-19 related reasons
15. I am concerned about the Australian economy
16. I am anxious about the healthcare system not being able to protect me or my loved ones
17. I am uneasy about returning to face-to-face interactions
18. I am stressed about my financial situation
19. I am concerned about society and social liberties degrading due to the pandemic

Scoring:
Concerns about catching COVID and infrastructure: items 1, 5, 6, 8, 10, 11, 12, 15, 16, 18, 19
Concerns about politics, liberties, economy: items 4, 7, 14, 17, 21
Financial concerns: items 2, 9, 20

COVID-19 Readiness
Please rate the extent to which each statement applies to you.
1=*strongly disagree*; 5=strongly agree

1. Should another lockdown be needed, I will follow the rules
2. Having another lockdown will violate my rights
3. If we have another lockdown, I can promptly adapt to the required lifestyle again
4. Mandatory COVID-19 vaccination requirements violate my civil rights
5. If we have another lockdown, I will break the rules
6. If we have another lockdown, I will be willing to adapt my behaviours
7. Suggestion of another lockdown should be met with utmost resistance
8. Having another lockdown will violate my rights (R)
9. Mandatory COVID-19 vaccination requirements violate my civil rights (R)
10. If we have another lockdown, I will break the rules (R)
11. Suggestion of another lockdown should be met with utmost resistance (R)

(R) = item reverse scored.

Scoring: mean score of all items

Compliance Attitudes
Rate the extent to which each statement applies to you currently.
Slider scale 0 (does not apply at all) to 100 (applies very much)

1. I am aware of the current COVID-19 rules or recommendations
2. I understand the current COVID-19 guidelines
3. I adhere to the current COVID-19 rules or recommendations
4. I trust the effectiveness of the current COVID-19 rules
5. I'm satisfied with the current COVID-19 rules or recommendations

Scoring: mean of all items

Please rate the extent to which each statement applied to you PRIOR to December 2021.

1. I was aware of the current COVID-19 rules or recommendations
2. I understood the COVID-19 guidelines

3. I adhered to the COVID-19 rules and recommendations
4. I trusted the effectiveness of the COVID-19 rules
5. I was satisfied with the past COVID-19 rules or recommendations

Scoring: mean of all items

Reasons for Vaccination
Please indicate how much you agree with each statement.
1=strongly disagree; 7=strongly agree

1. I got vaccinated to protect myself
2. I got vaccinated to protect the greater community
3. I got vaccinated to protect the vulnerable population
4. I got vaccinated to protect my family and friends
5. I got vaccinated because my work required it
6. I got vaccinated to regain freedoms (e.g., socialising, hospitality, events)
7. I got vaccinated to be able to travel
8. I got vaccinated because everyone else was getting vaccinated
9. I got vaccinated due to pressure from my family/friends
10. I got vaccinated due to pressures from my doctor(s)

Scoring:
To protect self and others: items 1, 2, 3, 4
External pressures: items 8, 9, 10
To regain freedoms: items 5, 6, 7

Reasons for Booster
Please indicate how much you agree with each statement.
1=strongly disagree; 7=strongly agree

Items given to vaccinated participants who had not yet received a booster:

1. I would get a COVID-19 booster dose: To protect myself
2. I would get a COVID-19 booster dose: To protect the greater community
3. I would get a COVID-19 booster dose: To protect the vulnerable population
4. I would get a COVID-19 booster dose: To protect my family and friends
5. I would get a COVID-19 booster dose: If my work required it
6. I would get a COVID-19 booster dose: To regain freedoms (e.g., socialising, hospitality, events)
7. I would get a COVID-19 booster dose: To be able to travel
8. I would get a COVID-19 booster dose: If everyone else was getting it
9. I would get a COVID-19 booster dose: Due to pressure from my family/friends
10. I would get a COVID-19 booster dose: Due to pressures from my doctor(s)

Items given to participants who had received a booster:

1. I got a COVID-19 booster dose: To protect myself
2. I got a COVID-19 booster dose: To protect the greater community
3. I got a COVID-19 booster dose: To protect the vulnerable population
4. I got a COVID-19 booster dose: To protect my family and friends
5. I got a COVID-19 booster dose: Because my work required it
6. I got a COVID-19 booster dose: To regain freedoms (e.g., socialising, hospitality, events)
7. I got a COVID-19 booster dose: To be able to travel
8. I got a COVID-19 booster dose: Because everyone else was getting it
9. I got a COVID-19 booster dose: Due to pressure from my family/friends
10. I got a COVID-19 booster dose: Due to pressures from my doctor(s)

Scoring:

To protect self and others: items 1, 2, 3, 4
External pressures: items 8, 9, 10
To regain freedoms: items 5, 6, 7

Trust
Please indicate your level of trust in each of the following:
1=*strongly distrust;* 5=*strongly trust*

1. Your State/Territory Government
2. Federal Government
3. Scientists
4. Mainstream Media
5. Health Care Professionals

How much do you trust each of these as sources of information about COVID-19?

6. Mainstream news (TV, radio, internet)
7. Official Government/health organization websites
8. Social media
9. Doctors
10. Other health professionals
11. Government agencies
12. Family or friends
13. Scientific articles
14. General public websites
15. Podcasts

Scoring:
Trust in science and health professionals: items 3, 5, 9, 10, 13
Trust in unofficial sources: items 8, 12, 14, 15
Trust in mainstream media: items 4, 6
Trust in government: items 1, 2, 7, 11

Exploratory Factor Analyses of Original Measures

COVID-19 Beliefs and Vaccination Attitudes Examination (VAX) Scale

Due to correlations between scores on the COVID-19 beliefs measure and the VAX Scale (ranging from -.20 to .79, $p < .01$) which caused multicollinearity issues in the model, exploratory factor analysis (EFA) was run on the items of both scales to examine their convergence. The analysis was run using Principal Axis Factoring with Promax rotation. The Kaiser criterion suggested six factors with eigenvalues greater than one, capturing 57% of variance. However, the scree plot indicated a possible three factor solution, and the fourth, fifth, and sixth factors captured only small (~2%) amounts of variance. Many items cross-loaded on multiple factors and some factors shared strong correlations approaching .70. Thus, this solution was not optimal, and we re-ran the analysis constraining the solution to 3, 4, and 5 factors. The amount of variance captured by each solution was 54.80% by 5 factors, 52.33% by 4 factors, and 49.50% by 3 factors. Examining the pattern matrices, the three-factor solution provided the most meaningful and interpretable factors with fewer cross-loadings than the four- and five-factor solutions. The scree plot also supported a three-factor solution. Thus, this was deemed the most suitable. The results are presented in Table C1. Mean scores were calculated based on which factor each item loaded highest on. Factor 1 was labelled Anti-Vax Beliefs, Factor 2 was labelled Beliefs in Protective Measures and Regulations, and Factor 3 was labelled Beliefs in Alternative Measures.

Table S2. Exploratory factor analysis of COVID-19 beliefs and Vaccination Attitudes Examination Scale (N=582).

Items	Factor Loadings			h^2
	1	2	3	
COVID-19 vaccines can cause unforeseen problems in children 12-16 years of age	**.93**	.26	-.04	.71
COVID-19 vaccines can cause unforeseen problems in children under 12 years of age	**.92**	.26	-.06	.63
I feel there are safe COVID-19 vaccines available	**-.92**	-.02	.11	.69
I worry about the unknown effects of COVID-19 vaccines in the future	**.89**	.29	.02	.41
I can rely on COVID-19 vaccines to stop serious symptoms and deaths from COVID-19	**-.84**	.08	.18	.56
The COVID-19 vaccine protects people	**-.82**	.11	.12	.59
There may be problems with the COVID-19 vaccines that we have not yet discovered	**.75**	.16	-.03	.57
COVID-19 vaccines are a big con (deception, fake)	**.74**	-.03	.07	.46
Authorities promote vaccination against COVID-19 for financial gain, not for people's health	**.65**	-.05	.08	.53
COVID-19 vaccines make a lot of money for pharmaceutical companies, but do not do much for regular people	**.60**	-.03	.10	.65
Mandatory COVID-19 vaccination requirements violate my civil rights	**.60**	-.20	.06	.45
Immunization of children under 16 years of age is unnecessary and problematic	**.59**	-.23	.03	.50
Anti-vaccination views should be a protected attribute under the Equal Opportunity Act (law to ensure employees are not unfairly disadvantaged)	**.58**	-.17	.11	.58
COVID-19 Vaccines are effective in slowing the spread of the pandemic	**-.56**	**.31**	.04	.46
Being exposed to COVID-19 naturally is safer for the immune system than being exposed through vaccination	**.55**	-.21	.09	.41
Immunization of children under 12 years of age is unnecessary and problematic	**.53**	-.23	.11	.46
Natural immunity lasts longer than a COVID-19 vaccination	**.52**	-.15	.08	.27
Discrimination law should protect everyone at their jobs who chooses not to get vaccinated (whether or not they have medical reasons preventing them from doing so)	**.50**	-.19	.15	.57
Natural exposure to the COVID-19 virus gives the safest protection	**.48**	-.21	.12	.60
Testing, tracking, and tracing every potential case of COVID-19 are effective measures to minimize the spread	.16	**.79**	.09	.52
Social distancing practices should remain in use until the COVID-19 pandemic is under control	.10	**.78**	-.01	.27
Lockdown policies are essential to keep in place until the COVID-19 pandemic is under control	.20	**.77**	-.01	.49
Wearing face masks in public places is effective in slowing the spread of COVID-19	.02	**.75**	-.05	.39
People should use QR codes when required	.01	**.71**	.02	.18
Social distancing is effective in slowing the spread of COVID-19	-.04	**.67**	.07	.53
Keeping enclosed spaces well-ventilated should be mandatory for schools, businesses and workplaces	.01	**.64**	.04	.44
Lockdowns are effective in slowing the spread of COVID-19	.04	**.64**	-.10	.60
Continuing hand hygiene is important in controlling the pandemic spread	-.08	**.59**	.14	.58
Ventilation in indoor areas (e.g., school, work, entertainment venues) is effective in slowing the spread of COVID-19	-.02	**.55**	.23	.27
Wearing masks for children is unnecessary and adds burden on children and parents	.08	**-.54**	.10	.56
Self-testing for COVID-19 by doing my own swab using TGA approved rapid test kits is effective in slowing the spread of the pandemic	-.17	**.45**	.16	.17
We should rely on people getting COVID-19 to build up (herd) immunity	.09	**-.30**	.24	.58
Social distancing and lockdown policies are detrimental to our economy	.09	-.23	.18	.45
Alternative medicine like consuming certain teas, herbs, minerals, or supplements are good preventative measures for COVID-19	-.04	.00	**.82**	.43
Taking a particular type of supplement/vitamin assists in recovering quickly if infected by COVID-19	-.02	.03	**.77**	.59
Taking a particular type of supplement/vitamin is effective in preventing getting infected by COVID-19	.05	.13	**.77**	.60
Inhaling steam e.g., with salts/oils should be used to prevent and to treat the COVID-19 infection	.06	.04	**.64**	.36
Oral and nasal hygiene (flossing and saline wash) should be recommended COVID-19 prevention measures	-.02	.25	**.45**	.63
Eigenvalues	14.65	3.55	1.99	
% of Variance	37.34	7.94	4.00	
Correlations				
2	-.64			
3	.59	.37		

Note. Factor loadings over |.30| are bolded.

Multidimensional COVID-19 Worry Index

An EFA (Principal Axis Factoring with Promax rotation) was run on the items of the Multidimensional COVID-19 Worry Index. The original scale had 21 items. One item "I am stressed about global economic recession" was removed as it cross-loaded and caused a Heywood case. Another item "I am calm and relaxed when I think about the pandemic" was removed as it was the only positively worded item. For the remaining items, the Kaiser criterion indicated four factors with eigenvalues greater than one, explaining 52.13% of the total variance. One item had cross-loadings on two factors and one item did not load strongly on any factor. Two factors correlated at .71, so the solution was re-run constraining the solution to three factors. The three-factor solution captured 49.16% of variance and was supported by the scree plot. All items loaded over .30 on one factor only and correlations between factors ranged from .48 to .52. Thus, this was deemed a better solution. The results are presented in Table C2. The first factor captures concerns about being infected by COVID-19 and about infrastructure not being able to cope (α = .90). The second factor captures concerns about political systems, social liberties, and the economy (α = .74). The third factor captures concerns about one's own financial and employment situation (α = .82). Mean scores were calculated for each factor based on these results.

Table S3. Exploratory factor analysis of the Multidimensional COVID-19 Worry Index (N=582).

Items	Factor Loadings			h^2
	1	2	3	
I am uneasy about returning to face-to-face interactions	**.76**	-.06	-.11	.48
I am concerned about the health of my family members due to COVID-19	**.74**	-.14	.06	.50
I am stressed about leaving my house	**.74**	-.02	.00	.53
I am nervous when I think about the pandemic	**.74**	-.11	.06	.51
I am worried about someone I love dying due to COVID-19 related reasons	**.71**	-.05	.04	.49
I am concerned about taking public transport due to COVID-19	**.69**	.01	-.10	.43
I am worried about my health due to COVID-19	**.68**	-.06	.07	.47
I am anxious about the healthcare system not being able to protect me or my loved ones	**.67**	.08	-.01	.50
I am stressed about our healthcare system being overloaded	**.62**	.19	-.05	.51
I am worried about pharmacies running out of medicines/essential health supplies	**.49**	.25	.07	.48
I am nervous about grocery stores running out of food and/or other supplies	**.38**	.27	.09	.39
I am worried about political unrest in Australia	.04	**.80**	-.11	.59
I am nervous about political systems failing	-.04	**.77**	-.06	.52
I am concerned about society and social liberties degrading due to the pandemic	-.11	**.61**	.08	.36
I am concerned about the Australian economy	-.03	**.49**	.16	.32
I am concerned about the possibility of another lockdown in the city/region where I live	-.04	**.33**	.20	.19
I am anxious about losing money due to COVID-19	-.06	-.03	**.97**	.86
I am worried about becoming unemployed due to the pandemic	-.01	.04	**.71**	.53
I am stressed about my financial situation	.15	.08	**.58**	.50
Eigenvalues	7.06	2.09	1.39	
% Of Variance	34.53	8.38	5.25	
Correlations				
2	.52			
3	.48	.48		

Note. Factor loadings over |.30| are bolded.

COVID-19 Readiness

An EFA (Principal Axis Factoring with Promax rotation) was run to examine the factorial structure of the COVID-19 readiness items. The Kaiser criterion indicated one factor with an eigenvalue over one, and this was clearly supported by the scree plot. This factor captured 54.68% of total variance and all items loaded highly and had good communalities. The results are presented in Table C3. Internal consistency was high with a Cronbach's alpha of .88.

Table S4. Exploratory factor analysis of COVID-19 readiness (N=582).

	Factor Loadings	h^2
If we have another lockdown, I will break the rules	.83	.69
Should another lockdown be needed, I will follow the rules	-.81	.66
Having another lockdown will violate my rights	.77	.59
Suggestion of another lockdown should be met with utmost resistance	.75	.56
If we have another lockdown, I will be willing to adapt my behaviours	-.74	.55
If we have another lockdown, I can promptly adapt to the required lifestyle again	-.65	.83
Mandatory COVID-19 vaccination requirements violate my civil rights	.60	.36

Compliance Attitudes

Two EFA's (Principal Axis Factoring with Promax rotation) were run to examine the structure of the compliance attitudes scale—one for the items asking participants to answer about how they feel at the present time, and one for the same items asking participants to retrospectively answer about how they felt during before December 2021. Firstly, for the 'current' items, the Kaiser criterion indicated two factors capturing 75% of variance, which correlated at .41. Given only two items loaded on factor two and the large amount of variance captured by the first factor, we also examined the results of constraining the solution to one factor. The one-factor solution captured 49.10% of variance. All items loaded strongly onto the factor and communalities were adequate, thus this solution was retained. Cronbach's alpha was .81. The factor loadings and communalities are presented in Table C4. For the 'before December 2021' items, similar results were obtained. Two factors captured 82.14% of variance, sharing a correlation of .52. One factor captured 58.84% of variance and communalities and factor loadings were high, so this solution was retained to allow for direct comparisons. Cronbach's alpha was .85. The results are presented in Table C5.

Table S5. Exploratory factor analysis of compliance attitudes (current) items (N=582).

	Factor Loadings	h^2
I understand the current COVID-19 guidelines	.83	.70
I am aware of the current COVID-19 rules or recommendations	.78	.61
I adhere to the current COVID-19 rules or recommendations	.67	.44
I trust the effectiveness of the current COVID-19 rules	.62	.38
I am satisfied with the current COVID-19 rules or recommendations	.58	.33

Table S6. Exploratory factor analysis of compliance attitudes (before December 2021) items (N=582).

	Factor Loadings	h^2
I understand the current COVID-19 guidelines	.83	.70
I am aware of the current COVID-19 rules or recommendations	.78	.61
I adhere to the current COVID-19 rules or recommendations	.67	.44
I trust the effectiveness of the current COVID-19 rules	.62	.38
I am satisfied with the current COVID-19 rules or recommendations	.58	.33

Trust

Items about trust in different institutions and authorities and items about trust in information sources correlated highly; thus, to avoid multicollinearity issues in the models, an EFA (Principal Axis Factoring with Promax rotation) was run to examine their convergence. There were four factors with eigenvalues greater than one, capturing 51.46% of variance. This solution was supported by the scree plot and factors were meaningful and interpretable, so this was deemed a good solution. All items loaded strongly on their respective factors, and communalities ranged from .22 to .70. The results are presented in Table C6. The first factor captures trust in health care professionals and scientists (α = .85). The second factor captures trust in unofficial sources of information (α = .71). The third factor captures trust in government (α = .72), and the fourth factor captures trust in the media (α = .82).

Table S7. Exploratory factor analysis of trust items (N=582).

Items	Factor Loadings				h^2
	1	2	3	4	
Information sources: A doctor	**.86**	.08	-.05	-.07	.64
Information sources: Other health professionals	**.83**	.20	-08	-.09	.58
Health Care Professionals	**.76**	-.11	-.07	.14	.62
Scientists	**.71**	-.15	-.06	.14	.56
Information sources: Scientific articles	**.70**	-.01	-.08	-.06	.40
Information sources: General public websites	.07	**.75**	-.02	.00	.56
Information sources: Podcasts	-.09	**.64**	-.04	.07	.45
Information sources: Social media	-.03	**.57**	.03	.11	.39
Information sources: Family or friends	.10	**.46**	.11	-.09	.22
Your State/Territory Government	-02	-.06	**.61**	.07	.38
Federal Government	-.28	.10	**.60**	-.03	.26
Information sources: Official Government/health organisation websites	**.33**	-.07	**.60**	.02	.70
Information sources: Government agencies	.3	.07	**.58**	-.03	.62
Mainstream Media	.00	-.01	-.03	**.81**	.62
Information sources: Mainstream news (TV, radio, internet)	.01	.12	.07	**.74**	.70
Eigenvalues	4.8	2.55	1.27	1.03	
% Of Variance	29.28	13.57	5.04	3.58	
Correlations					
2	-.03				
3	.59	.23			
4	.40	.35	.57		

Vaccination Reasons

An EFA (Principal Axis Factoring with Promax rotation) was run on the items about reasons for getting vaccinated. The Kaiser criterion suggested three factors capturing 62.79% of variance. The scree plot also clearly indicated three factors. The interpretability of these factors was clear with no cross-loadings and small correlations between each factor, so thus solution was retained. The results are presented in Table C7. The first factor captures getting vaccinated to protect self and others (α = .92). The second factor captures getting vaccinated due to external pressures from others (α = .74). The third factor captures getting vaccinated to regain freedoms e.g., socializing, travel, and work (α = .69). Communalities were generally high, ranging from .24 to .90 and correlations ranged from .16 to .30.

Table S8. Exploratory factor analysis of vaccination reasons (N=554).

	Factor loadings			h^2
	1	2	3	
I got vaccinated to protect the greater community	**.94**	.00	.02	.90
I got vaccinated to protect the vulnerable population	**.91**	.03	-.02	.82
I got vaccinated to protect my family and friends	**.91**	.00	-.02	.82
I got vaccinated to protect myself	**.69**	-.04	.01	.48
I got vaccinated due to pressure from my family/friends	-.07	**.92**	-.04	.85
I got vaccinated due to pressures from my doctor(s)	.02	**.71**	-.05	.48
I got vaccinated because everyone else was getting vaccinated	.09	**.50**	.28	.41
I got vaccinated to regain freedoms (e.g., socialising, hospitality, events)	-.06	-.07	**.85**	.68
I got vaccinated to be able to travel	.04	-.03	**.77**	.59
I got vaccinated because my work required it	-.01	.19	**.40**	.24
Eigenvalues	3.38	2.38	1.32	
% Of Variance	31.61	21.49	9.69	
Correlations				
2	-.17			
3	.16	.30		

Note. Factor loadings over |.30| are bolded.

Booster Reasons

An EFA (Principal Axis Factor with Promax rotation) was run on booster reasons. People who had already received a booster were asked regarding their reasons for getting a booster (n=274), and vaccinated people who had not yet received a booster were asked about the reasons they would get a booster (n=280). The Kaiser criterion and scree plot indicated three factors, and the solution was identical to that for the vaccination reason items, thus this solution was retained. The three factors captured 67.73% of the total variance collectively. The first factor captures getting a booster to protect self and others (α = .89). The second factor captures getting a booster due to external pressures from others (α = .75). The third factor captures getting a booster to regain freedoms e.g., socializing, travel, and work (α = .70). The results are presented in Table C8. All items loaded strongly onto their respective factors and communalities were generally high, ranging from .27 to .92.

Table S9. Exploratory factor analysis of booster reasons (N=554).

Items	Factor Loadings			h^2
	1	2	3	
To protect the greater community	**.95**	-.01	.03	.92
To protect the vulnerable population	**.94**	.04	-.02	.87
To protect my family and friends	**.92**	.04	-.01	.84
To protect myself	**.70**	-.08	-.02	.51
Due to pressure from my family/friends	-.06	**.94**	-.05	.88
Due to pressures from my doctor(s)	.01	**.81**	-.05	.63
If everyone else was getting it/Because everyone else was getting it	.08	**.53**	.25	.44
To regain freedoms (e.g., socialising, hospitality, events)	-.02	-.07	**.97**	.90
To be able to travel	.03	.00	**.71**	.52
If my work required it/Because my work required it	-.06	.23	**.40**	.27
Eigenvalues	3.23	2.49	1.05	
% Of Variance	32.33	24.88	10.52	
Factor Correlations				
2	-.06			
3	.17	.34		

Note. Factor loadings over |.30| are bolded.

Data Quality Checking Protocol

A total of 598 people completed the survey. To ensure good quality data, the following checks were used to identify careless responders:

1. Five bogus items (Huang et al., 2015; Meade & Craig, 2012)
   - Table D1 presents bogus items, criteria for failing check, and percentage of respondents flagged for failing each item.

2. Five logic checks (Wardropper et al., 2021)
   - Five items were added to check for logically inconsistent responding. These items were identical to original survey items except for the addition of a negative adverb (e.g., not) to invert the meaning. Table D2 presents logic check items and percentage of respondents flagged for failing each check. Logic check items were all rated on a 6-point Likert scale (1=strongly disagree; 2=somewhat disagree; 3=disagree; 4=somewhat agree; 5=agree, 6=strongly agree). The logic for determining inconsistent responding was that if response to item 1 was 1 to 3 (somewhat to strongly disagree) then the response to Item 2 should be 4 to 6 (somewhat to strongly agree), and vice versa. That is, it would be logically inconsistent to either agree or disagree to both items in a pair, and respondents who did were flagged.

Table D3 presents frequencies for total number of flags received by participants. Respondents with three or more flags in total were removed from the sample. A total of 16 participants (2.6%) received three or more flags and were excluded.

Participants were also screened based on completion time. The median completion time of all participants (N=598) was 36.25 minutes. Nine people were identified as fast completers, defined by completing in less than half the median, and were reviewed case by case. The two fastest completers were unvaccinated so were not required to complete a large portion of the survey; thus, their completion time was justified. All had circumstances which meant some questions were skipped for them e.g., have no children, had not done any COVID-19 tests, or had no vaccination symptoms. Thus, they were left in the sample. Five did not fail any of the bogus or logic checks, two failed one check, and two participants failed two.

Table S10. Bogus items

Item	Responses	Frequencies	"Incorrect" response	% Flagged
I have been to every country in the world (Meade & Craig, 2012)	1=strongly disagree	533 (89.1%)	3, 4, 5, 6, 7	4.9
	2=disagree	36 (6%)		
	3=somewhat disagree	7 (1.2%)		
	4=neither agree nor disagree	11 (1.8%)		
	5=somewhat agree	6 (1.0%)		
	6=agree	1 (0.2%)		
	7=strongly agree	4 (0.7%)		
I sleep less than one hour per night (Meade & Craig, 2012)	1=very inaccurate	550 (92%)	3, 4, 5	2.8
	2= moderately inaccurate	31 (5.2%)		
	3=neither inaccurate nor accurate	9 (1.5%)		
	4=moderately accurate	6 (1%)		
	5=very accurate	2 (0.3%)		
I do not understand a word of English (Meade & Craig, 2012)	1=strongly disagree	569 (95.2%)	3, 4, 5	3.3
	2=disagree	9 (1.5%)		
	3=neither agree nor disagree	14 (2.3%)		
	4=agree	4 (0.7%)		
	5=strongly agree	2 (0.3%)		
I have never brushed my teeth (Meade & Craig, 2012)	1=very inaccurate	551 (92.1%)	3, 4, 5	3.7
	2= moderately inaccurate	25 (4.2%)		
	3=neither inaccurate nor accurate	9 (1.5%)		
	4=moderately accurate	12 (2%)		
	5=very accurate	1 (0.2%)		
I have never used a computer in my life (Huang et al., 2015)	1=strongly disagree	575 (96.2%)	3, 4, 5, 6, 7	2.8
	2=disagree	7 (1.2%)		
	3=somewhat disagree	1 (0.2%)		
	4=neither agree nor disagree	9 (1.5%)		
	5=somewhat agree	1 (0.2%)		
	6=agree	1 (0.2%)		
	7=strongly agree	4 (0.7%)		

Table S11. Logic checks

Item 1 (original item)	Item 2	Frequency flagged
I feel there are safe COVID-19 vaccines available	I do not feel there are safe COVID-19 vaccines available	1 (0.2%)
I worry about the unknown effects of COVID-19 vaccines in the future	I am not worried about the unknown effects of COVID-19 vaccines in the future	67 (11.2%)
COVID-19 vaccines protect people	COVID-19 vaccines do not protect people	0
Natural immunity lasts longer than a COVID-19 vaccination	Natural immunity does not last longer than a COVID-19 vaccination	26 (4.3%)
I can rely on COVID-19 vaccines to prevent serious symptoms and death from COVID-19	I cannot rely on COVID-19 vaccines to prevent serious symptoms and death from COVID-19	14 (2.3%)

Table S12. Frequencies for number of flags received by participants in total.

Number of total flags	Frequency
0	436 (72.9%)
1	120 (20.1%)
2	26 (4.3%)
3	5 (0.8%)
4	5 (0.8%)
5	6 (1.0%)

Sample Characteristics

Table S13. Sample characteristics (N=582)

Variable	Level	Frequency	Sample %	Population %[1]
Sex	Male	239	41.1	49.1
	Female	333	57.2	50.9
Age	18-29 years	258	44.3	21.1
	30- 39 years	145	24.9	18.8
	40-49 years	90	15.4	16.5
	50-59 years	58	10.0	15.6
	60-69 years	26	4.3	13.5
	70 years or older	5	0.9	14.7
State	ACT	14	2.4	1.7
	NSW	175	30.1	32.0
	NT	3	0.5	1.0
	QLD	107	18.4	20.1
	SA	46	7.9	7.2
	TAS	13	2.2	2.2
	VIC	165	28.4	25.3
	WA	59	10.1	10.6
Country of birth	Australia	395	67.9	71.7
Citizenship	Australian citizen	497	85.4	82.4
Aboriginal or Torres Strait Islander	Yes	4	0.7	2.8
Marital status	Married (registered) or living with someone in a relationship	282	48.4	48.1
	Separated but not divorced	7	1.2	3.2
	Divorced	26	4.5	8.5
	Widowed	3	0.5	5.2
	Never married and not living with someone in a relationship	264	45.4	35.0
Highest level of education	Year 11 or below	12	2.1	21.2
	Year 12	116	19.9	15.7
	Trade certificate/apprenticeship	44	7.6	24.3
	Diploma	51	8.8	10.1
	Bachelor's degree	225	38.7	16.0
	Higher degree	134	23.0	12.7
In general, would you say that your health is:	Excellent	54	9.3	9.9
	Very good	181	31.1	34.2
	Good	223	38.3	36.9
	Fair	97	16.7	15.2
	Poor	27	4.6	3.7
Chronic conditions	Asthma, emphysema, or chronic bronchitis	68	11.7	n/a
	Arthritis or rheumatism	23	4.0	20.5
	Cancer diagnosed in last 3 years	6	1.0	n/a
	Diabetes	14	2.4	6.2
	Digestive problems (e.g., stomach ulcer, colitis, gallbladder disease)	34	5.8	7.4
	Heart trouble (e.g., angina, congestive heart failure, coronary artery disease)	17	2.9	n/a
	HIV illness or AIDS	0	0	n/a
	Kidney disease	1	0.2	1.2

Liver problems (e.g., cirrhosis	7	1.2	n/a
Stroke	1	0.2	0.8
None of these	457	78.5	n/a

[1]Age and sex values were obtained from national, state and territory population data, Australian Bureau of Statistics, published June, 2021: https://www.abs.gov.au/statistics/people/population/national-state-and-territory-population/latest-release. Population values for State, Country of Birth, Australian Citizenship, Aboriginal or Torres Strait Islander Status and Marital Status were obtained from the Australian Bureau of Statistics 2016 Census (note, this data was not limited to those aged 18 and over). Population values for Highest Level of Education and the General Health Question were derived from the Household, Income and Labour Dynamics in Australia Survey (HILDA, Wave 19), limited to those aged 18 and over. Prevalence of chronic conditions were derived from the Australian Bureau of Statistics National Health Survey, published December, 2018: https://www.abs.gov.au/statistics/health/health-conditions-and-risks/national-health-survey-first-results/latest-release. Population data for other chronic conditions was not directly comparable in format.

Post-Hoc Analyses on Health Literacy

Table S14. ANOVAs and pairwise comparisons between the three profiles in health literacy subscales

	Mean (SD)			ANOVA			Pairwise comparisons		
	Class 1 (n = 176)	Class 2 (n = 353)	Class 3 (n = 53)	*F*	*p-value*	η^2	c1-2	c1-3	c2-3
	hesitant-vaxxers	pro-vaxxers	anti-vaxxers						
Functional	4.80 (1.04)	5.00 (0.98)	4.87 (0.94)	2.43	.09	0.03	.08	.90	.60
Critical	4.66 (2.16)	4.92 (1.99)	5.96 (1.79)	10.02	<.001	0.12	.40	<.001	<.001
Communicative	4.61 (1.63)	5.18 (1.25)	5.13 (1.24)	8.33	<.001	0.11	<.001	.04	.97
